# Lightweight Semantic-Aware Route Planning on Edge Hardware for Indoor Mobile Robots: Monocular Camera–2D LiDAR Fusion with Penalty-Weighted Nav2 Route Server Replanning

**DOI:** 10.3390/s26072232

**Published:** 2026-04-04

**Authors:** Bogdan Felician Abaza, Andrei-Alexandru Staicu, Cristian Vasile Doicin

**Affiliations:** Faculty of Industrial Engineering and Robotics (FIIR), National University of Science and Technology POLITEHNICA Bucharest, 060042 Bucharest, Romania; andrei.staicu@stud.fiir.upb.ro (A.-A.S.); cristian.doicin@upb.ro (C.V.D.)

**Keywords:** AMR, semantic navigation, Nav2 route server, camera-LiDAR fusion, edge computing, Raspberry Pi 5, YOLO, mobile robot, ROS 2

## Abstract

**Highlights:**

**What are the main findings?**

- A lightweight, deterministic camera–2D LiDAR fusion method (Angular Sector Fusion, ASF) enables robust object localization in the map frame on CPU-only edge hardware, with minimal computational overhead and no learned fusion parameters.
- Penalty-weighted semantic routing with the Nav2 Route Server is feasible and reliable on physical robots, enabling online replanning based on runtime-updated semantic penalties and achieving high success rates across multi-robot, multi-session trials.

**What are the implications of the main findings?**

- Semantic information can be injected at the route level (graph routing) without replacing Nav2’s proven execution stack, providing a practical pathway from perception to route decisions that is deployable on low-cost platforms.
- The paper provides reproducible deployment patterns and workarounds for the Nav2 community (graph export constraints, route execution edge cases, and configuration details), supporting the faster adoption of semantic-aware routing in real ROS 2 systems.

**Abstract:**

The paper introduces a computationally efficient semantic-aware route planning framework for indoor mobile robots, designed for real-time execution on resource-constrained edge hardware (Raspberry Pi 5, CPU-only). The proposed architecture fuses monocular object detection with 2D LiDAR-based range estimation and integrates the resulting semantic annotations into the Nav2 Route Server for penalty-weighted route selection. Object localization in the map frame is achieved through the Angular Sector Fusion (ASF) pipeline, a deterministic geometric method requiring no parameter tuning. The ASF projects YOLO bounding boxes onto LiDAR angular sectors and estimates the object range using a 25th-percentile distance statistic, providing robustness to sparse returns and partial occlusions. All intrinsic and extrinsic sensor parameters are resolved at runtime via ROS 2 topic introspection and the URDF transform tree, enabling platform-agnostic deployment. Detected entities are classified according to mobility semantics (dynamic, static, and minor) and persistently encoded in a GeoJSON-based semantic map, with these annotations subsequently propagated to navigation graph edges as additive penalties and velocity constraints. Route computation is performed by the Nav2 Route Server through the minimization of a composite cost functional combining geometric path length with semantic penalties. A reactive replanning module monitors semantic cost updates during execution and triggers route invalidation and re-computation when threshold violations occur. Experimental evaluation over 115 navigation segments (legs) on three heterogeneous robotic platforms (two single-board RPi5 configurations and one dual-board setup with inference offloading) yielded an overall success rate of 97% (baseline: 100%, adaptive: 94%), with 42 replanning events observed in 57% of adaptive trials. Navigation time distributions exhibited statistically significant departures from normality (Shapiro–Wilk, *p* < 0.005). While central tendency differences between the baseline and adaptive modes were not significant (Mann–Whitney U, *p* = 0.157), the adaptive planner reduced temporal variance substantially (σ = 11.0 s vs. 31.1 s; Levene’s test W = 3.14, *p* = 0.082), primarily by mitigating AMCL recovery-induced outliers. On-device YOLO26n inference, executed via the NCNN backend, achieved 5.5 ± 0.7 FPS (167 ± 21 ms latency), and distributed inference reduced the average system CPU load from 85% to 48%. The study further reports deployment-level observations relevant to the Nav2 ecosystem, including GeoJSON metadata persistence constraints, graph discontinuity (“path-gap”) artifacts, and practical Route Server configuration patterns for semantic cost integration.

## 1. Introduction

Autonomous indoor navigation for mobile robots has matured substantially with the widespread adoption of the Robot Operating System 2 (ROS 2) and its Navigation 2 (Nav2) stack [1,2]. Modern platforms routinely achieve reliable point-to-point navigation using occupancy grid maps, adaptive Monte Carlo localization (AMCL) [2], and model-predictive controllers, such as MPPI [3]. However, standard Nav2 deployments treat the environment primarily as a static geometric space: the costmap encodes obstacles as binary cells, and the planner optimizes for the shortest distance without a semantic understanding of what those obstacles represent [1].

This geometric-only view becomes insufficient when the robot must reason about the navigational significance of detected objects. A person standing near a corridor junction, a chair partially blocking a path, or a fragile object on the floor all present fundamentally different navigation requirements that cannot be captured by costmap cell costs alone. Semantic awareness—the ability to detect, classify, and reason about objects in the environment—enables a robot to make route-level decisions, such as preferring a longer but unobstructed path over a shorter path that passes through a cluster of people [4,5].

Recent work has addressed this gap through semantic mapping approaches that fuse camera-based object detection with range sensing to build object-level environment representations [5,6,7]. However, most of these systems stop at map construction: they annotate the environment with object locations but do not close the loop to influence navigation decisions. The Nav2 Route Server [8], introduced as a graph-based alternative to free-space planners, provides a natural integration point for semantic information through its edge metadata and scoring plugin architecture [8,9,10]—yet its use with runtime semantic penalties has not been experimentally validated on physical robots. In other words, the missing piece is a reproducible perception-to-route loop that updates routing decisions online, under real sensing noise and localization drift, on CPU-only edge hardware.

A further practical challenge is that many semantic navigation systems require GPU-equipped platforms or powerful onboard computers, limiting their applicability to low-cost, energy-constrained edge platforms such as the Raspberry Pi 5. Deploying real-time object detection (YOLO family) [11,12] alongside the full Nav2 stack on a single ARM-based board requires careful engineering of inference pipelines and CPU budgeting, often using optimized inference backends such as NCNN [13]. Therefore, evaluation must report not only navigation outcomes but also deployment metrics (inference rate, latency, and CPU utilization) to demonstrate feasibility under real-time constraints.

This paper addresses these challenges with three contributions:

**C1—Angular Sector Fusion (ASF):** A lightweight, geometric method for fusing monocular camera bounding boxes with 2D LiDAR range measurements to localize detected objects in the map frame. ASF operates with zero learned parameters, auto-configures from standard ROS 2 topics and the URDF transform tree (consistent with REP-105 frame conventions) [14,15], and adds less than 5% CPU overhead at a 3 Hz fusion rate on the RPi5.

**C2—Experimental validation of semantic routing with the Nav2 Route Server:** A controlled ablation evaluation (baseline vs. adaptive) on physical mobile robots, demonstrating penalty-weighted route selection with online replanning under real sensing noise and localization uncertainty. We validate the system on 115 navigation legs across three robot platforms (including two distinct compute architectures), demonstrating 42 replanning events and a 97% success rate.

**C3—Practical deployment characterization:** A detailed documentation of deployment constraints encountered during Route Server integration on edge hardware, including the GeoJSON metadata limitation, the path-gap phenomenon, and the use start requirement—providing reproducible deployment patterns and workarounds that can be directly reused by the Nav2 community [8,9,10,15].

The three robot platforms (Xplorer-A, B, and C) used in this work were designed and assembled in-house within the university’s robotics research teams, following a project-based pedagogy approach that integrates research and education [16]. Beyond hardware construction, all three robots run an author-controlled baseline stack (motor control and odometry via ROS 2 Control, IMU and LiDAR integration, and state estimation), implemented and validated across three physical units; this reduces hidden software confounders when comparing baseline versus adaptive navigation and increases confidence that observed differences are attributable to the proposed semantic routing layer [10].

Details of the baseline stack and its interfaces are summarized in Section 3. Section 3 provides a module-level system description (ROS interfaces and persisted artifacts) to support reproducibility and implementation traceability. The remainder of this paper is organized as follows. Section 2 reviews related work in semantic mapping, camera–LiDAR fusion, and Nav2 extensions. Section 3 describes the system architecture, including the ASF pipeline, the persistent semantic map, navigation graph annotation, and Route Server integration. Section 4 presents the experimental methodology. Section 5 reports the results. Section 6 discusses the findings, compares them with related work, and identifies limitations. Section 7 concludes and outlines future work.

## 2. Related Work

This section reviews prior work in (i) semantic mapping for mobile robot navigation, (ii) camera–LiDAR fusion for object localization, (iii) Nav2 Route Server and graph-based planning extensions, and (iv) deployment of semantic perception on edge hardware within ROS 2 systems.

### 2.1. Semantic Mapping for Mobile Robot Navigation

Semantic mapping extends metric environment representations with object-level information, providing robots with a richer understanding of their surroundings. Kostavelis and Gasteratos [17] provided a comprehensive survey of semantic mapping approaches, highlighting the progression from purely geometric maps to representations that encode object identities, spatial relationships, and functional attributes. More recently, semantic navigation has been increasingly framed as an end-to-end system problem (perception, representation, and action selection), motivating systematic reviews focused specifically on semantic navigation pipelines and their integration into navigation behaviors [12].

In practical mobile robotics stacks, semantic mapping is often realized by integrating deep learning object detectors, most commonly from the YOLO family [11,12], with SLAM/localization pipelines so that detections can be anchored consistently in a global map frame. Alqobali et al. [7] demonstrated a semantic SLAM system for indoor mobile robots that fuses YOLOv3 object detection with 2D LiDAR measurements on a Raspberry Pi 4. Their system projects detected bounding box centers to LiDAR bearings and estimates object distance from the nearest LiDAR return. While this work validated the feasibility of camera–LiDAR semantic fusion on low-cost hardware, it was limited to simulation (Gazebo), used a single robot, and did not incorporate semantic information into the navigation planner—detected objects were placed on the map but did not influence route selection.

A key direction in the literature is therefore not only producing semantic maps but enabling planning decisions based on semantic state, as already motivated in earlier foundational work on using semantic maps for task planning [6]. Our work extends this line of research in three directions: (1) we introduce Angular Sector Fusion, which uses the full angular sector rather than a single bearing for more robust distance estimation; (2) we close the perception-to-planning loop by feeding semantic penalties into a graph-based routing layer (Nav2 Route Server) for penalty-weighted route selection; and (3) we validate the approach on physical hardware across three robots with two distinct compute architectures, using an author-controlled full-stack ROS 2 baseline previously validated on real robots [18].

### 2.2. Camera–LiDAR Fusion for Object Localization

Fusion of camera and LiDAR data for object localization spans a spectrum from deep learned approaches to geometric projection methods. Deep fusion methods such as PointPainting [19] and Frustum PointNets [20] achieve high accuracy but typically rely on 3D LiDAR point clouds and GPU computation, making them unsuitable for low-cost 2D-LiDAR edge platforms where the CPU budget is constrained. At the lightweight end of the spectrum, geometric projection methods exploit the known spatial relationship between the camera and LiDAR to associate detections with range measurements.

The simplest geometric approach projects the bounding box center to a single LiDAR bearing and reads the corresponding range value. This method is computationally trivial but sensitive to false associations when objects do not align with the exact center bearing—a common situation with wide or off-center bounding boxes. More robust geometric approaches project the full bounding box to an angular sector and aggregate multiple LiDAR returns within that sector to derive a stable distance estimate. Recent Sensors work also supports the value of efficient camera–LiDAR fusion strategies that are compatible with computational constraints and do not require full 3D point cloud processing [21].

Our ASF method follows this lightweight geometric family but adds two properties that are important for deployable robotic systems: (i) it is fully deterministic (no learned fusion parameters), and (ii) it self-configures at runtime from the standard ROS topics and the TF2, enabling portability across robots with different sensor mounting geometries without source code changes (see Section 3 and the REP-105 conventions [14,15]).

### 2.3. Nav2 Route Server and Graph-Based Planning

ROS 2 and Nav2 have become a widely adopted baseline for autonomous indoor navigation, supported by extensive documentation and standardized lifecycle patterns [1,2]. In Nav2, most semantic navigation integrations target the local costmap layer or the behavior-tree logic, where semantics modulate local obstacle avoidance but do not necessarily influence higher-level route selection [1]. For route-level decision-making, a graph-based representation offers a natural abstraction: nodes represent key places and edges represent traversable connections whose costs can encode semantic preferences.

The Nav2 Route Server [9] was introduced as a graph-based alternative to free-space global planning. It exposes a ComputeRoute interface and a scoring architecture where plugins can modify edge costs based on metadata, enabling route selection based on user-defined constraints [4,10]. This creates a direct mechanism to inject semantic penalties into routing decisions (e.g., preferring corridors that are semantically “safer” or less congested), while preserving Nav2’s proven execution stack (AMCL, controllers, and costmap-based local avoidance). However, the published literature contains limited experimental validation of Route Server operation under runtime-updated semantic penalties on physical robots, particularly on CPU-only edge hardware where perception and routing must coexist within a tight compute envelope.

In our work, the Route Server integration is specifically engineered for online semantic replanning: a prescan phase establishes semantic penalties, the Route Server is reloaded with an annotated graph, and an execution loop monitors penalty deltas and triggers rerouting when changes exceed a threshold. The approach is implemented within the documented Route Server architectures and operations [9], while also addressing practical deployment constraints observed in the ecosystem (e.g., plugin metadata typing constraints and Route Server tooling limitations) [22,23].

### 2.4. Edge Deployment of Neural Network Inference in ROS 2 Navigation

A recurring barrier for semantic navigation in practice is the computational cost of real-time perception. Many semantic navigation systems assume GPUs or high-power embedded computers, which is incompatible with low-cost edge platforms. In contrast, modern ROS 2 deployments increasingly target lightweight compute (ARM SBCs) due to cost, energy, and integration constraints. Achieving robust performance on such platforms requires both efficient inference backends and a careful ROS 2 execution architecture [14].

Within this work, YOLO-family detectors provide a strong accuracy–speed tradeoff for real-time object detection [11,12], and optimized inference runtimes such as NCNN enable CPU-only deployment on ARM platforms [13]. At the system level, ROS 2 middleware choices and configurations can also affect latency and determinism. eProsima Fast DDS, for example, is commonly evaluated as a high-performance RMW option in ROS 2 distributions [24]. In addition, planning and control components must remain computationally stable during perception bursts; thus, we base navigation execution on established Nav2 components (e.g., Smac planners and the MPPI controller) [4,25] and their configuration constraints [23]. For reproducibility and the calibration of expected performance envelopes, public benchmarking datasets and comparisons of Nav2 controllers provide useful baselines [26].

These constraints motivate the evaluation approach adopted in this paper: beyond navigation success and trajectory metrics, we report deployment-level metrics such as the inference rate, latency, and CPU load, ensuring that the semantic-aware routing layer is demonstrated as feasible under realistic edge compute budgets.

## 3. System Architecture

This section describes the system architecture in detail, organized to reflect the paper’s contributions: C1 is detailed in Section 3.2 and Section 3.3 (ASF + persistent semantic map), C2 in Section 3.4 and Section 3.5 (graph annotation + Route Server integration), and C3 is supported by the practical integration constraints and workarounds documented throughout Section 3.4 and Section 3.5 (dual-graph conversion, path-gap handling, and Route Server operation constraints). The source code for all modules described in this section, together with the automated test framework and the complete experimental dataset (115 navigation legs across 27 sessions), is publicly available in the repository referenced in the Data Availability Statement.

The navigation baseline relies on ROS 2 and Nav2 components following standard lifecycle patterns and documented configuration interfaces [1,3,4]. The route-level planning is implemented through the Nav2 Route Server and the nav2_route package, which expose graph-based routing via ComputeRoute and related tools [9,10,22].

Regarding the robotic platforms and baseline stack (custom full-stack), all three experimental platforms (Xplorer robots A–C) are custom-built differential-drive mobile robots running a fully custom ROS 2 stack developed by the authors, including the ROS 2 Control hardware interface, wheel-encoder odometry, IMU integration, and a dedicated sensor-fusion pipeline for state estimation. This baseline stack was implemented from scratch to ensure consistent timing, interface compatibility, and repeatable navigation behavior across the three physical units, providing a reliable foundation for evaluating the proposed semantic-aware routing layer, while respecting standard ROS coordinate-frame conventions and transformation setup guidelines [14,15].

### 3.1. Platform Description

**Figure 1:**
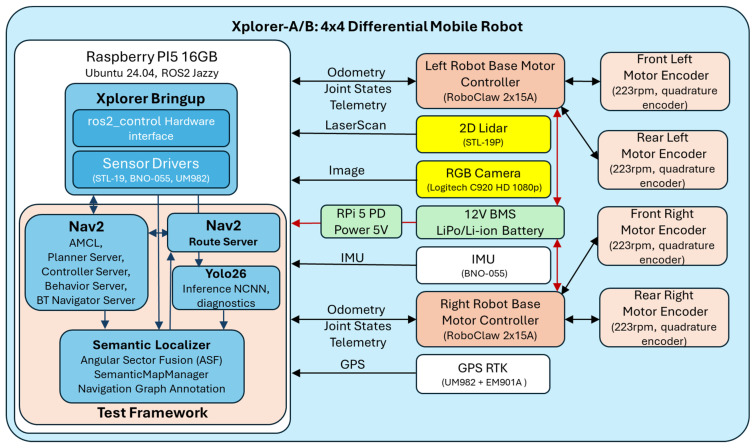
System architecture of the Xplorer A and B differential-drive mobile robots. Software components (left, blue) run on a Raspberry Pi 5 (16 GB). The Nav2 Route Server [3,16] operates as a standalone lifecycle node, separate from the main Nav2 navigation stack. The Yolo26 detector publishes both object detections and inference diagnostics. The Semantic Localizer fuses camera and LiDAR data to annotate the navigation graph with semantic penalties.

The generic experimental platform is a four-wheel-drive differential mobile robot (Xplorer) designed for indoor navigation research. All computation runs on a single Raspberry Pi 5 (Raspberry Pi Ltd, Cambridge, England) with 16 GB, ARM Cortex-A76, Ubuntu 24.04 without GPU or NPU acceleration (Figure 1). This design reflects a typical edge deployment scenario where perception and navigation must share a tight compute envelope [14,26]. The platform integrates a Logitech C920 HD monocular camera (Logitech, Lausanne, Switzerland) with natively 1080 p, configured at 640 × 480 to maximize the frame rate, a 2D LiDAR STL-19P/LD19 (LDROBOT, Shenzhen, China) with 360° field of view, approximately 503 rays at 10 Hz, and a BNO055 (I2C) inertial measurement unit (Bosch Sensortec GmbH, Reutlingen, Germany). The drivetrain comprises four DC motors with quadrature encoders (223 rpm) controlled by two RoboClaw 2 × 15A motor controllers (Basicmicro Motion Control, Temecula, CA, USA) communicating over UART in packet serial mode. Power is supplied by a 12 V lithium-ion battery pack with an integrated battery management system, with the RPi5 powered through USB Power Delivery at 5 V.

The software stack is built on ROS 2 Jazzy with Nav2 1.3.10. The navigation stack includes AMCL for localization against a pre-built occupancy grid, an Extended Kalman Filter fusing wheel odometry and IMU data at 20 Hz, the MPPI controller at 20 Hz for local trajectory optimization, and SmacPlannerHybrid (Reeds-Shepp model) for global path planning in the baseline condition. The Nav2 Route Server [9] operates as a standalone lifecycle node, deliberately kept outside the Nav2 lifecycle manager to avoid interference with the proven navigation stack.

**Figure 2:**
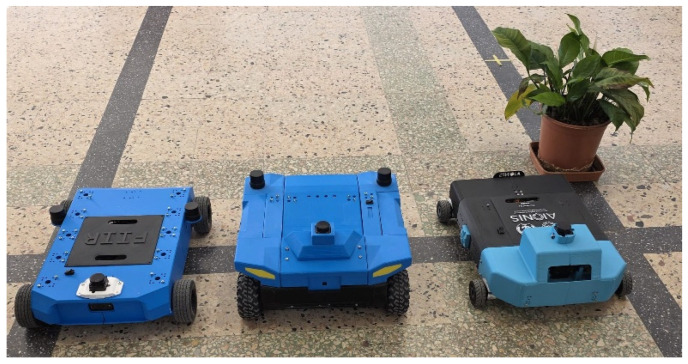
Xplorer robots A (**left**), B (**center**), and C (**right**).

Based on the generic experimental platform, three robots (designated Xplorer-A, Xplorer-B, and Xplorer-C) are used in the experiments (Figure 2). All three platforms were designed and built in-house within the university’s robotics research teams, following a project-based pedagogy approach [16]. Xplorer-A and Xplorer-B share identical hardware configurations (single RPi5, Table 1). Xplorer-C uses a different drivetrain geometry but the same sensor suite (C920, STL-19P, and BNO055) and a dual-RPi5 architecture: the first RPi5 runs the complete Nav2 navigation stack, EKF, AMCL, and the semantic localizer, while the second RPi5—connected via Ethernet in a local ROS 2 DDS network—runs exclusively the yolo26_cpp detector node. This split-compute configuration offloads YOLO inference from the navigation processor, enabling the evaluation of the system under reduced CPU contention. From the perspective of the semantic pipeline, the architecture is transparent: detection messages arrive on the same `’/yolo26/detections’ topic regardless of whether the detector runs locally or on a remote node, and the ASF pipeline auto-configures identically in both cases. This three-robot configuration enables cross-platform validation of the perception and planning pipeline across two distinct compute architectures (single-board vs split-inference) and different drivetrain geometries.

Object detection is performed by a custom C++ ROS 2 package (yolo26_cpp) that wraps YOLO26 inference [6] using the NCNN framework [13], following the broader YOLO design lineage for real-time object detection [5]. NCNN was selected over the ONNX Runtime and OpenCV’s DNN module for its ARM-specific optimizations, including NEON SIMD intrinsics and memory packing layouts that are particularly effective on the Cortex-A76 cores of the RPi5. The detector is compiled with aggressive optimization flags (‘-O3 -march = native -mtune = native -ffast-math’) and implements the ROS 2 managed (lifecycle) node pattern: model loading occurs at the ‘configure’ transition (a one-time operation of approximately 2 s), while the camera subscription and inference loop are activated at the `activate` transition. This separation ensures that the computationally expensive model initialization does not delay the navigation stack startup. The detector, which is configured with 4 inference threads (one per Cortex-A76 core), processes frames resized to 416 × 416 pixels (the NCNN model input resolution), regardless of the camera’s native output resolution.

A software rate limiter regulates the detection frequency at 15 Hz, dropping incoming camera frames when they arrive faster than the configured rate. Since the camera publishes at approximately 30 fps, this policy halves the detector’s CPU load while maintaining sufficient temporal resolution for the 3 Hz semantic fusion pipeline downstream. The detector publishes object detections as ‘vision_msgs/Detection2DArray’ on the topic ‘/yolo26/detections’ and, since version v5.10 of the test framework, also publishes inference diagnostics (FPS, inference time in milliseconds, and detection count) as a JSON-formatted ‘std_msgs/String’ on the topic ‘/yolo26/diagnostics’ at 5 s intervals. The diagnostics topic incurs zero computational overhead when no subscribers are present, as the publication is gated by ‘get_subscription_count()’.

Table 2 lists only the interfaces directly used by the proposed semantic routing pipeline and by the experimental framework. Standard ROS 2/Nav2/ros2_control interfaces (e.g., TF, costmap topics, controller internals) are omitted for brevity. All three platforms (Xplorer A–C) run a fully author-developed baseline stack (ROS 2 Control hardware interface, odometry, IMU/LiDAR integration, and state estimation), providing a controlled foundation for evaluating the proposed semantic-aware routing layer. The source code for all modules listed in Table 2 is available in the repository referenced in the Data Availability Statement.

### 3.2. Angular Sector Fusion Pipeline

**Figure 3:**
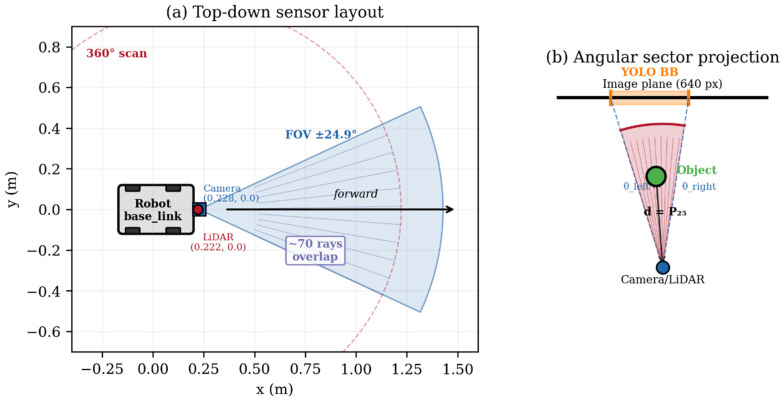
Geometric relationship between the monocular camera and 2D LiDAR on the Xplorer platform. (**a**) Top-down view showing co-located sensors. (**b**) Angular overlap: the camera’s horizontal FOV (±24.9°) is covered by approximately 70 LiDAR rays at 0.72° angular resolution. Each detected bounding box maps to a sector of LiDAR rays from which the object distance is estimated.

The core perception method localizes objects detected by the camera in the robot’s map frame by fusing 2D bounding box geometry with LiDAR range measurements (Figure 3). We refer to this approach as Angular Sector Fusion (ASF) to distinguish it from deep sensor fusion methods that operate on raw feature representations. The ASF is a geometric, closed-form method with no learned parameters, making it fully deterministic and computationally lightweight.

#### 3.2.1. Sensor Geometry and Runtime Auto-Configuration

A key design principle of the ASF pipeline is that no sensor-specific geometric constants are hardcoded. All parameters are acquired at runtime from standard ROS 2 topics and the TF2 transform tree, enabling the pipeline to operate without modification across robots with different sensor models, mounting positions, or URDF descriptions. The TF2 transforms are queried and applied consistently with REP-105 frame conventions and Nav2 transformation setup guidelines [14,15].

The camera intrinsic parameters—focal length f__x_ and principal point c__x_—are extracted once from the first ‘sensor_msgs/CameraInfo’ message received on ‘/camera/camera_info’. These values are populated automatically by the camera driver based on the physical sensor characteristics (for the Logitech C920 at 640 × 480: f__x_ = 687.54 px, c__x_ = 308.11 px). The LiDAR scan geometry—angular range, angular increment, range limits, and the number of rays—is similarly extracted from the first ‘sensor_msgs/LaserScan’ message on ‘/scan’ (for the STL-19P: 503 rays at a 0.716° increment over 360°). The spatial relationship between the camera, LiDAR, and robot base frames is resolved dynamically through the TF2 lookups using the frame IDs specified as ROS parameters (base_laser’, ‘camera_link_optical’, and ‘map’), which are defined in each robot’s URDF.

This runtime self-configuration means that deploying the pipeline on a different robot requires only that the new platform publish the standard ROS topics and provide a valid URDF—no source code changes are needed. We validated this property across three robots (Xplorer-A, B, andC) with different URDF descriptions and confirmed identical pipeline behavior.

#### 3.2.2. Pipeline Steps

The ASF pipeline executes the following steps for each YOLO detection message, rate-limited to 3 Hz to preserve the CPU budget:

**Step 1—Bounding box to camera angles.** For each detected object, the horizontal extent of the bounding box (pixel columns x__min_ and x__max_) is converted to angular coordinates in the camera frame using the pinhole projection model:

θ__left_ = atan2(x__min_ − c__x_, f__x_)

θ__right_ = atan2(x__max_ − c__x_, f__x_)

where f__x_ and c__x_ are the focal length and the principal point acquired at runtime from the CameraInfo topic (see Table 3). These parameters are sensor-specific and adapt automatically when the camera model or the resolution changes, without requiring any code modification.

**Step 2—Camera angles to LiDAR sector.** The camera angles are mapped to the LiDAR angular reference frame. Since the sensors are co-located on the same forward axis (lateral offset = 0, longitudinal offset = 6 mm, negligible for objects beyond 0.5 m), the mapping reduces to a sign inversion plus a configurable yaw correction:

θ__lidar_min_ = −θ__right_ + θ__offset_

θ__lidar_max_ = −θ__left_ + θ__offset_

where θ__offset_ (default 0.0 rad) compensates for any residual angular misalignment between the camera and the LiDAR optical axes. The LiDAR ray indices corresponding to these angles are computed using the angular increment from the LaserScan message:

idx = (θ − angle__min_)/angle__increment_

This calculation adapts automatically to any 2D LiDAR regardless of its angular range or resolution.

**Step 3—LiDAR distance extraction.** The LiDAR rays within the angular sector (θ__lidar_min_, θ__lidar_max_) are selected from the most recent scan. Invalid measurements (out of range or non-finite) are filtered. The 25th percentile of the valid range measurements is taken as the estimated distance to the object:

d = percentile__25_(valid_ranges)

The 25th percentile was chosen rather than the mean or the median because the nearest valid surface within the bounding box sector is most likely to correspond to the detected object, while farther returns may originate from background surfaces. A minimum of two valid rays is required for a measurement to be accepted (configurable via ‘min_valid_rays’).

**Step 4—Polar to Cartesian transformation.** The distance and the center angle are converted to a Cartesian point in the LiDAR frame:

x__laser_ = d · cos(θ__center_)

y__laser_ = d · sin(θ__center_)

where θ__center_ is the angular midpoint of the LiDAR sector.

**Step 5—Coordinate frame transformation.** The point in the LiDAR frame (base laser) is transformed to the global map frame using the TF2 library. The transform chain base_laser → base_link → odom → map is resolved dynamically from the robot’s URDF (for static transforms) and AMCL (for the map→odom transform). The frame identifiers are ROS parameters, not hardcoded strings, thus allowing deployment on platforms with different frame naming conventions.

A critical implementation detail is the use of a zero timestamp (‘Time (seconds = 0, nanoseconds = 0)’) for the TF lookup, which retrieves the most recent available transform regardless of the timestamp. This is necessary because AMCL publishes the map→odom transform with a variable delay of 1–5 s relative to the scan timestamp; using the scan’s own timestamp or the current clock would cause extrapolation errors.

**Step 6—Temporal tracking and stabilization.** Each localized object is matched to an existing tracked object by the class name and spatial proximity (Euclidean distance < 0.5 m). If matched, the new position is appended to a sliding window buffer. The stable position is computed as the element-wise median of the last W positions (default W = 5):

x__stable_ = median(x__{t-W + 1}_, ..., x__t_)

y__stable_ = median(y__{t-W + 1}_, ..., y__t_)

The median filter provides robustness against outlier measurements caused by transient occlusions or LiDAR multipath reflections. An object is considered stable and eligible for map persistence after a configurable minimum number of detections (default: 3). Objects that are not re-detected within the tracking timeout (5 s) are removed from the active tracking list.

#### 3.2.3. Computational Considerations

**Figure 4:**
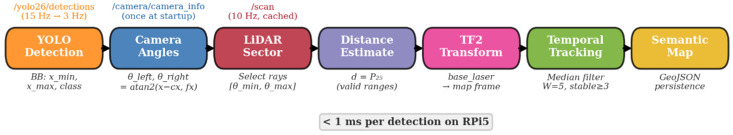
Angular Sector Fusion (ASF) pipeline. The YOLO bounding boxes are projected to angular sectors in the LiDAR frame. The 25th percentile of the valid LiDAR ranges provides the estimated distance. The resulting polar coordinates are transformed to the global map frame via the TF2, followed by the temporal median filtering for position stabilization.

The entire ASF pipeline—from bounding box parsing through the TF lookup to the tracking update—executes in under 1 ms per detection on the RPi5, dominated by the TF2 lookup latency (Figure 4). At the configured rate of 3 Hz with typically 1–5 detections per frame, the semantic localizer node consumes approximately 2–5% of the total CPU. On the single-RPi5 platforms (Xplorer-A and B), the combined system load—YOLO detector (~10–15% CPU at 5.5 Hz effective rate), the Nav2 stack (~55–60%), and the semantic pipeline (~5%)—totals approximately 85%, leaving a margin for transient load spikes during the MPPI optimization or the AMCL particle filter updates. On Xplorer-C, where the YOLO inference runs on the dedicated second RPi5, the navigation processor operates at approximately 48% CPU, providing substantial headroom for additional computational tasks.

Implementation details enabling robustness on physical robots. Two engineering choices were critical for reliable operation under AMCL latency and sensor asynchrony: (i) the TF2 transforms are queried using a zero timestamp to always retrieve the latest available map→odom transform, thus avoiding extrapolation failures under AMCL’s variable publication delay; and (ii) the object range is estimated using a lower percentile of valid LiDAR rays inside the bounding-box sector, which biases the estimate toward the nearest consistent surface and reduces background contamination when the sector intersects distant walls. These choices allow the ASF to remain deterministic and lightweight while being robust to typical indoor sensing artifacts.

#### 3.2.4. Operating Envelope

The ASF pipeline assumes that detected objects have a physical extent that intersects the 2D LiDAR scan plane. On the Xplorer platforms, the LiDAR is mounted at approximately 16 cm above ground level; therefore, common indoor objects such as persons, chairs, benches, potted plants, and bottles reliably produce LiDAR returns within the bounding-box angular sector. Objects located entirely above the scan plane (e.g., wall-mounted signs, ceiling-hung obstacles) or entirely below it (e.g., flat floor markings) will be detected by the camera but will not receive a valid range estimate, causing them to be filtered out at Step 3 (minimum two valid rays requirement). This constraint is inherent to any 2D LiDAR fusion approach and is addressed in future work through the integration with 3D depth cameras (Section 7).

### 3.3. Persistent Semantic Map

Objects localized by the ASF pipeline are stored in a persistent semantic map managed by the SemanticMapManager module. This module maintains an in-memory dictionary of objects and periodically serializes them to a GeoJSON FeatureCollection on disk (‘semantic_objects.geojson’), enabling recovery across node restarts.

#### 3.3.1. Mobility Classification and TTL

Each detected object class is categorized by its expected mobility in an indoor environment (Table 4):

The base penalty values reflect the navigation significance of each category: dynamic objects (primarily people) receive the highest penalty to strongly encourage route avoidance, while minor objects receive a lower but still meaningful penalty. The speed limit is expressed as a percentage of the robot’s maximum velocity, following the Nav2 Route Server convention [8,9].

#### 3.3.2. Object Matching and Position Fusion

When a new detection arrives from the ASF pipeline, the manager searches the existing object store for a match based on two criteria: an identical class name and a Euclidean distance below a configurable threshold (default: 0.5 m). If a match is found, the update strategy depends on the mobility category. For dynamic objects, the position is overwritten directly, since these objects are expected to move. For static and minor objects, an exponential moving average (EMA) fusion is applied:

x__updated_ = α · x__existing_ + (1 − α) · x__new_

y__updated_ = α · y__existing_ + (1 − α) · y__new_

where α = 0.7 weights the existing position more heavily, providing stability against measurement noise while allowing gradual position refinement as more observations accumulate. The confidence score is updated to the maximum observed value, and the observation count is incremented.

#### 3.3.3. TTL-Based Cleanup

A periodic cleanup timer (every 10 s) removes objects whose time since the last observation exceeds their TTL. Dynamic objects (persons) expire after 60 s without re-observation, reflecting the assumption that people move away. Minor objects expire after 120 s. Static objects (furniture) are permanent and never expire. When objects are removed, the corresponding edge annotations in the navigation graph are updated at the next save cycle, causing the Route Server to re-evaluate routes through the affected area.

#### 3.3.4. Persistence Format

Objects are serialized as a GeoJSON FeatureCollection where each Feature is a Point with coordinates in the map frame and properties including the class name, mobility category, confidence, observation count, and timestamps. This format is human-readable, compatible with GIS tools (QGIS), and easily parsed by external analysis scripts. Disk writes occur every 30 s and on the node shutdown, balancing data safety against the I/O overhead on the RPi5’s SD card/Disk storage.

This plays a role in the experimental protocol: in the adaptive condition, the prescan phase populates this persistent map, after which the object entries (subject to TTL and stabilization rules) are used to annotate the navigation graph and trigger Route Server reloading; the return legs reuse the stored map without repeating the prescan, ensuring that the routing behavior is driven by the same semantic state established at the start of the run.

### 3.4. Navigation Graph Annotation

**Figure 5:**
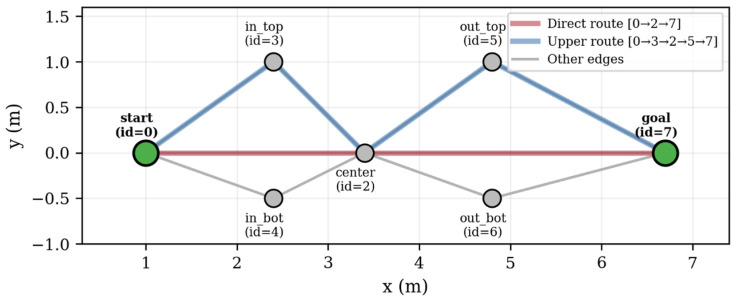
Navigation graph for the FIIR corridor experiment. Seven nodes define two alternative routes between the start (node 0) and the goal (node 7): an upper route through nodes 3–2–5 and a lower route through nodes 4–2–6.

The semantic map is linked to the navigation topology through the automatic annotation of graph edges. The navigation graph is a GeoJSON file containing nodes (Point features with unique IDs and map frame coordinates) and directed edges (LineString features with start/end node IDs) as it is described in Figure 5. The graph used in our experiments contains 7 nodes and 20 directed edges (10 bidirectional pairs), forming a connected topology with two primary alternative routes between the start and goal positions and a direct central path (Figure 5). The central node (node 2) serves as a hub connecting to six of the other nodes, providing route flexibility. The edge annotations show the semantic penalties computed from the detected objects. The Route Server selects the route with the lowest total cost (distance + weighted penalty).

#### 3.4.1. Penalty Computation

For each edge in the graph, the system computes the minimum distance from every persistent object to the edge segment using the standard point-to-segment distance formula. Objects within a configurable proximity radius (default: 1.5 m) contribute to the edge’s aggregate penalty:

penalty__edge_ = Σ__i_ (P__base_(mobility__i_) × proximity_factor__i_ × confidence__i_)

where

proximity__factor_ = max(0.1, 1.0 − d__object_to_edge_/d__proximity_)

This formulation ensures that objects closer to the edge contribute proportionally higher penalties, while objects at the edge of the proximity radius contribute a minimum of 10% of their base penalty. The confidence term further modulates the penalty, reducing the influence of uncertain detections.

The speed limit for each edge is set to the minimum across all nearby objects:

speed_limit__edge_ = min__i_(S(mobility__i_))

where S returns 30%, 60%, or 90% depending on the mobility category (Table 4).

#### 3.4.2. Nav2 GeoJSON Compatibility

A critical practical constraint discovered during the integration is that the Nav2 Route Server’s GeoJsonGraphFileLoader (version 1.3.10) accepts only scalar numeric values in edge metadata. Any nested objects, arrays, or string values cause a ‘bad any_cast’ error at configuration time, because the scoring plugins attempt ‘any_cast<double>()’ on all metadata keys. To address this, the system produces two versions of the annotated graph:

1. Full semantic graph (‘route_graph_fiir_semantic.geojson’): contains complete metadata including the penalty, speed limit, class labels, and an array of nearby objects with details. This file serves as a diagnostic and analysis resource.
2. Nav2-compatible graph (‘route_graph_fiir_nav2.geojson’): contains only ‘penalty’ (float) and ‘speed_limit’ (float) in edge metadata, stripped of all non-numeric fields. This file is loaded by the Route Server.

The conversion is performed internally by the ‘save_nav2_graph()’ method, eliminating the need for an external conversion script. At the test framework startup, the Nav2-compatible graph is regenerated automatically if it is missing or older than the source graph.

This matters (practical contribution) because this dual-graph mechanism turns a Route Server constraint into a reproducible deployment pattern: the semantic graph preserves rich diagnostics for analysis and debugging, while the Nav2-compatible graph guarantees runtime stability of the scoring pipeline under strict metadata typing. This approach removes the need for external conversion scripts and ensures that semantic routing can be updated repeatedly at runtime without restarting the navigation stack.

### 3.5. Nav2 Route Server Integration

The Nav2 Route Server provides graph-based route planning as an alternative to free-space planners [8,9,10]. In our architecture, it operates as a standalone lifecycle node, deliberately separated from the Nav2 lifecycle manager that controls AMCL, the planner server, and the controller server. This separation ensures that the Route Server can be configured, activated, and reloaded independently without affecting the proven navigation stack.

**Figure 6:**
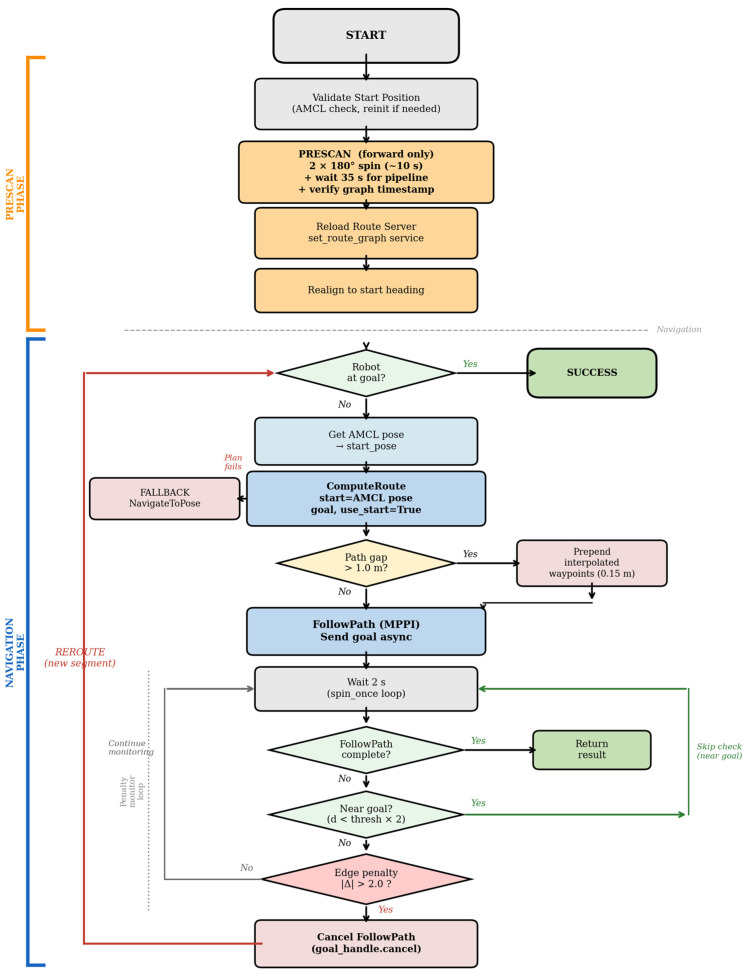
Adaptive navigation flowchart for the forward leg.

In Figure 6 the pre-scan phase (left bracket, orange) performs a 360° environmental scan, waits for the semantic pipeline to annotate the navigation graph, reloads the Route Server, and realigns the robot. The navigation phase (left bracket, blue) executes an outer loop of route segments: ComputeRoute selects the lowest-cost route from the current AMCL position, a path-gap correction is applied if needed, and FollowPath (MPPI) executes the dense path. An inner penalty monitoring loop checks edge penalties every 2 s; if any penalty change exceeds δ = 2.0, FollowPath is canceled and a new route is computed (reroute). If the robot is near the goal, penalty checks are skipped. If route planning fails, the system falls back to NavigateToPose.

#### 3.5.1. Architecture

We use the ComputeRoute-to-FollowPath architecture, where the Route Server computes a dense path that is executed directly by the MPPI controller without an intermediate global planner. This is combined with the AdjustSpeedLimit operation, which publishes speed limits on the ‘/speed_limit’ topic based on the semantic metadata of edges along the current route. This architecture is documented as Architecture #1 and Operation #5 in the Nav2 Route Server documentation [9].

The ComputeRoute action accepts start and goal poses and returns a dense path sampled at 0.05 m intervals along with the selected route expressed as node IDs and edge IDs. The MPPI controller follows this dense path directly via the FollowPath action, without invoking an intermediate global planner. This direct execution is justified by the small graph size (7 nodes, maximum edge length approximately 2 m) and the indoor environment, where MPPI’s local obstacle avoidance from the costmap is sufficient to handle minor deviations from the planned path. The AdjustSpeedLimit operation reads the `speed_limit` metadata from edges along the active route and publishes the corresponding velocity constraint, allowing the controller to reduce speed near semantically significant objects.

Runtime semantic replanning is triggered as follows: during execution, the system monitors edge penalties at a fixed interval and triggers replanning when a change larger than a threshold δ is observed on any edge belonging to the current route; this creates a lightweight closed loop between perception-driven map updates and route selection without requiring continuous global re-planning.

#### 3.5.2. Scoring Configuration

Route selection is determined by two edge scoring plugins operating in combination. The DistanceScorer (weight = 1.0) evaluates each edge by its geometric length, scaled by the ‘speed_limit’ metadata when present. The PenaltyScorer (weight = 5.0) reads the ‘penalty’ value from edge metadata and adds it to the edge cost. The 5:1 weight ratio ensures that semantic penalties dominate route selection when objects are present. As a concrete example, a person detected near an edge contributes a penalty of approximately 50 × proximity_factor × confidence ≈ 25–40 after scaling (Table 4), which translates to 125–200 weighted cost units—easily outweighing the typical distance cost of 1–3 units for a 1–2 m edge.

#### 3.5.3. Dynamic Graph Reload

The Route Server supports runtime graph replacement through the ‘set_route_graph’ service. The Semantic Localizer invokes this service after each periodic save cycle (every 30 s) to reload the updated Nav2-compatible graph. Additionally, the test framework explicitly triggers a graph reload after the prescan phase (Section 3.6) to ensure that newly detected objects are reflected in the next route computation. This mechanism enables quasi-dynamic semantic routing: the environment is scanned, the graph is updated, and subsequent route requests account for the current object layout. The term “quasi-dynamic” is deliberate—true real-time dynamic routing would require continuous graph updates during navigation, whereas our implementation updates periodically and on-demand.

### 3.6. Adaptive Navigation with Replanning

The adaptive navigation mode (Test 3 in the experimental protocol) combines environment perception with semantic route planning in a structured two-phase sequence, illustrated in Figure 6.

#### 3.6.1. Prescan Phase

Before each forward navigation leg, the robot performs a 360° environmental scan consisting of two consecutive 180° rotations using the Nav2 Spin action, totaling approximately 10 s. This is followed by a configurable waiting period (default: 35 s) during which the Semantic Localizer processes the accumulated detections, updates the persistent map, annotates the navigation graph with penalties, and generates the Nav2 compatible GeoJSON file. The framework confirms that the graph file has been updated on disk by checking its modification timestamp, then explicitly reloads the Route Server via the ‘set_route_graph’ service. Finally, the robot realigns to the starting heading to ensure a consistent initial orientation for the navigation phase.

The prescan is performed only for forward legs (start → goal). Return legs (goal → start) do not repeat the prescan, relying instead on the persistent semantic map populated during the forward prescan—objects detected during the forward scan remain in memory and continue to influence graph penalties for the return navigation. This design halves the prescan overhead per round trip while maintaining semantic awareness in both directions.

The prescan duration (rotation + wait + realignment) is excluded from the reported navigation time to enable a fair comparison with the baseline condition, which does not perform a prescan. The total prescan overhead is reported separately in the experimental results.

#### 3.6.2. Navigation with Penalty Monitoring

The navigation phase is managed by the ‘exec_follow_with_replan()’ method, which implements a two-level loop structure (Figure 6, blue bracket).

At the outer level, each iteration computes a new route segment. The method first checks whether the robot has reached the goal (Euclidean distance below the node proximity threshold of 0.5 m), terminating with success if so. Otherwise, it obtains the robot’s current position from the AMCL pose and issues a ComputeRoute request with ‘use_start = True’ and the explicit AMCL coordinates as the start pose. Using an explicit start pose rather than allowing the Route Server to determine the position from TF is essential after AMCL reinitialization, when the map → odom transform may not yet have converged and would produce a stale or incorrect start position.

If the dense path returned by the Route Server begins more than 1.0 m from the robot’s current position—a known behavior when the nearest-node search assigns the robot to a non-adjacent graph node—interpolated waypoints are prepended at 0.15 m intervals from the robot to the first path point, ensuring that the MPPI controller receives a continuous path. Without this path-gap correction, the controller terminates the path immediately, reporting success despite the robot not having moved. If route planning fails entirely, the system falls back to NavigateToPose with SmacPlannerHybrid to ensure the robot reaches its goal regardless of Route Server availability.

At the inner level, the method monitors semantic penalties while the MPPI controller follows the current path. Every 2 s, the penalty values are read from the Nav2-compatible GeoJSON file on disk and compared against the values recorded when the current route was computed. If any edge on the active route has changed by more than a threshold δ = 2.0, the current FollowPath action is canceled, and control returns to the outer loop, which computes a new route from the robot’s updated position. This constitutes a reroute event, logged with full details including the previous and new route nodes, costs, penalties, and timing. An early termination guard skips the penalty check when the robot is within twice the node proximity threshold of the goal, preventing unnecessary replanning during the final approach.

#### 3.6.3. Baseline Condition

The baseline condition (Test 2 in the experimental protocol) uses Nav2’s standard NavigateToPose action with SmacPlannerHybrid for global path planning and MPPI for local trajectory control. No semantic information, no Route Server, and no prescan are involved. The costmap provides the only obstacle avoidance mechanism. This configuration serves as a clean reference for evaluating the effect of semantic awareness on navigation performance: any difference in route selection, navigation time, or proximity to objects between the baseline and adaptive conditions is attributable to the semantic pipeline and Route Server integration.

## 4. Experimental Methodology

### 4.1. Environment and Navigation Graph

The experiments were executed on three physical robots (Xplorer A–C) with identical software architecture and version-pinned components, where the complete base stack (motor control, odometry, IMU/LiDAR integration, and state estimation) is author-developed to guarantee repeatability across units.

**Figure 7:**
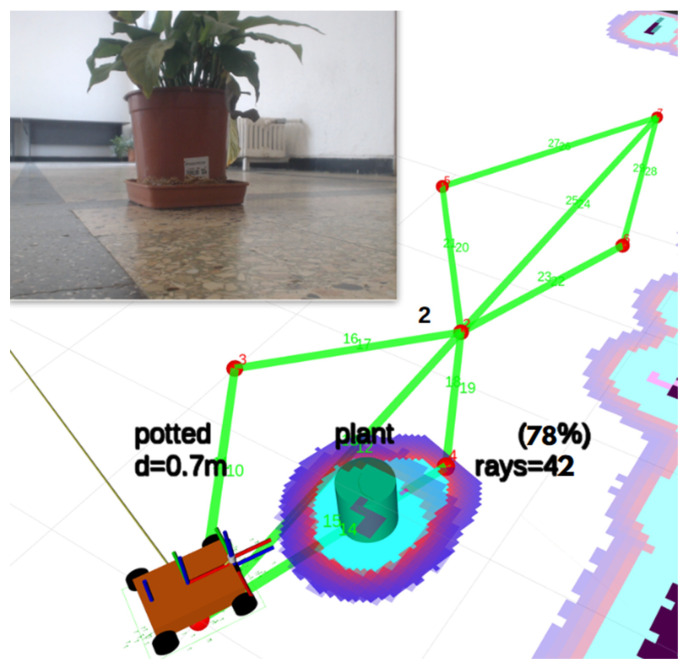
Experimental environment: FIIR second-floor corridor with the 7-node navigation graph overlaid. The central node (node 2) serves as a hub connecting to six of the other nodes. Objects detected by the semantic pipeline are shown as colored markers.

Experiments were conducted in the second-floor corridor of the Faculty of Industrial Engineering and Robotics (FIIR) at the National University of Science and Technology POLITEHNICA Bucharest (Figure 7). The environment is a furnished indoor corridor approximately 10 × 10 m, mapped as a 2D occupancy grid using SLAM Toolbox [27,28]. The corridor contains typical indoor objects including chairs, benches, potted plants, bottles, and occasionally people passing through.

The navigation graph consists of 7 nodes and 20 directed edges (10 bidirectional pairs), encoded as a GeoJSON file following the Nav2 Route Server format. The graph defines two primary alternative routes between the start (node 0) and goal (node 7) positions, plus a direct central path (Table 5).

Three alternative routes exist from start to goal:

- Upper: 0 → 3 → 2 → 5 → 7, passing through nodes (3, 2, 5)
- Lower: 0 → 4 → 2 → 6 → 7, passing through nodes (4, 2, 6)
- Direct: 0 → 2 → 7, through the central hub only

Route classification is performed automatically by the ‘identify_route()’ function, which computes the minimum distance from the executed path to each alternative’s characteristic nodes. If the path passes within 0.5 m of at least one characteristic node, that alternative is marked as active. When multiple alternatives are active (e.g., the path passes through nodes from both routes), it is classified as MIX.

### 4.2. Test Protocol

All experiments are managed by a custom automated test framework (v5.10, approximately 2050 lines of Python 3.12), fully parameterized through a YAML configuration file (‘route_config.yaml’). The framework requires no manual intervention between runs—it handles AMCL reinitialization, heading alignment, prescan execution, route computation, navigation, data collection, and result validation autonomously.

The experimental protocol comprises four test phases, executed in sequence according Table 6.

Each run in Tests 2 and 3 consists of a forward leg (start → goal) and a return leg (goal → start). Between legs, the robot performs a heading alignment spin to face the return direction, and AMCL is reinitialized at the current terminal node to prevent localization drift accumulation across runs.

#### 4.2.1. Experimental Design and Sample Size

The evaluation follows a controlled ablation study with a repeated-measures structure. The independent variable is the navigation mode: baseline (Nav2 NavigateToPose with SmacPlannerHybrid + MPPI, no semantic map, no Route Server, no prescan) versus adaptive (semantic-aware planning via Route Server with penalty-weighted edges and replanning). The experimental unit is a navigation leg, defined as a single autonomous traversal from start to goal (forward) or goal to start (return) under a fixed route graph and controller configuration.

Blocking and repetition are handled as follows: to reduce confounding and to capture real-world variability, trials are repeated across three robot platforms and multiple sessions/days, which act as blocking factors (robot and session/day). Within each run, forward and return legs are executed with AMCL reinitialization at terminals to limit drift accumulation across repetitions.

Sample size rationale is as follows: the total number of legs was chosen to balance practical constraints of real indoor corridor experiments (battery autonomy, safety, and naturally varying human traffic) with sufficient replication to estimate central tendency and dispersion of navigation and planning metrics across robots and sessions. In total, 115 navigation legs (45 baseline, 70 adaptive) were collected, and validated runs were used for performance summaries as described in Section 4.3.

##### Test 2—Baseline Protocol

For each leg, the baseline protocol:

1. Validates the robot position relative to the expected start node (threshold: 2.0 m); triggers AMCL reinitialization if needed.
2. Issues a NavigateToPose action to the goal node coordinates. SmacPlannerHybrid computes a global path; MPPI follows it with local obstacle avoidance from the costmap.
3. No semantic information, no Route Server, and no prescan are involved.

##### Test 3—Adaptive Semantic Protocol

For each forward leg, the adaptive protocol:

1. **Prescan phase:** Two consecutive 180° rotations (~7–10 s total) to expose all objects within the LiDAR/camera FOV, followed by a configurable waiting period (default: 35 s) for the semantic pipeline to process detections, update the persistent map, annotate the navigation graph, and generate the Nav2-compatible GeoJSON. Graph update on disk is confirmed by a timestamp check. The Route Server is explicitly reloaded via the ‘set_route_graph’ service. The robot realigns to the starting heading.
2. **Navigation phase:** ‘exec_follow_with_replan()’ issues a ComputeRoute from the current AMCL position (‘use_start = True’) to the goal, then follows the resulting dense path via FollowPath. Every 2 s, edge penalties are read from disk; if any edge on the current route has changed by more than δ = 2.0, FollowPath is canceled and a new ComputeRoute is issued from the current position. If route planning fails, the system falls back to NavigateToPose.
3. **Path gap correction:** If the dense path returned by Route Server begins more than 1.0 m from the robot’s current position (a known behavior when the nearest-node lookup assigns the robot to a non-adjacent graph node), interpolated waypoints are prepended at 0.15 m intervals from the robot to the path start, ensuring the MPPI receives a continuous path.

For return legs, the prescan is omitted (objects detected during the forward prescan remain in the persistent map), and navigation proceeds directly with ‘exec_follow_with_replan()’.

Timing separation: The prescan duration (scan + wait + realignment) is recorded separately from the navigation duration. The reported navigation time reflects only the active movement phase, enabling a fair comparison with the baseline condition. The total duration (prescan + navigation) is also reported for completeness.

### 4.3. Validation Criteria

A navigation leg is classified as a validated success if all four conditions are met:

1. The Nav2 action server reports success (goal reached)
2. The distance traveled ≥ 1.0 m (rejects false positives where MPPI terminates instantly)
3. The XY position error at goal ≤ 2.0 m (AMCL pose vs target coordinates)
4. The navigation duration ≥ 3.0 s (rejects premature termination)

These thresholds are configured in ‘route_config.yaml’ and were determined empirically based on the graph dimensions (start-to-goal distance: approximately 5.7 m straight line, 6–7 m along the graph) and the localization accuracy of the AMCL on the test platforms.

The maximum number of consecutive failures before aborting a test is configurable (default: 2), preventing extended test sessions when systematic issues (e.g., AMCL drift, costmap corruption) make further runs unreliable.

The validated-runs policy is as follows: all aggregated performance tables and comparisons in Section 5 use validated runs only to ensure consistent evaluation conditions and to prevent protocol-level failures (e.g., premature action termination or localization resets) from biasing navigation performance statistics.

### 4.4. Metrics

The test framework collects metrics at multiple levels, all persisted automatically as JSON files per run and aggregated in a session summary:

Metrics are computed per navigation leg and aggregated per robot and condition to support repeated-measures comparisons across blocked sessions (Table 7).

System metrics are collected at 1 Hz in a background thread throughout each navigation leg and stored as a time series in the JSON output, enabling post hoc analysis of CPU load profiles during navigation. YOLO inference diagnostics (FPS, inference time, detection count) are published by the detector node at 5 s intervals on ‘/yolo26/diagnostics’ and captured by the same background collector.

### 4.5. Reproducibility

The entire experimental configuration is captured in two files: ‘route_config.yaml’ (graph topology, terminal nodes, route alternatives, validation thresholds, timing parameters) and the GeoJSON graph file (node coordinates, edge connectivity). A session directory is created automatically for each test run, containing the session configuration snapshot, semantic map backup, all per-run JSON results, and the aggregated summary. No manual transcription of results is required.

Three robots (Xplorer-A, Xplorer-B, Xplorer-C) execute the same test protocol on the same navigation graph. Xplorer-A and B share an identical single-RPi5 hardware (Table 1), while Xplorer-C uses a dual-RPi5 architecture with YOLO inference offloaded to a dedicated compute unit connected via Ethernet. The only additional difference is the drivetrain geometry on Xplorer-C, which results in a different URDF description. This heterogeneous configuration enables a cross-platform comparison along two axes: (1) navigation and perception consistency across platforms with identical compute but different mechanical designs (A vs. B vs. C), and (2) the effect of inference offloading on CPU utilization and navigation reliability (A/B vs. C). The runtime self-configuration of the ASF pipeline (Section 3.2) handles the different URDF descriptions and compute topologies without code modification.

## 5. Results

Experiments were conducted across three robot platforms executing the automated test protocol described in Section 4. The evaluation follows a controlled ablation study with a repeated-measures structure: the only experimental manipulation is the navigation mode (baseline vs. adaptive), while trials are repeated across robots and sessions (blocking factors) under the same route graph and controller configuration. A total of 115 navigation legs (45 baseline, 70 adaptive) were collected, of which 111 met the validation criteria in Section 4.3 (97%). The complete dataset, organized as JSON files per run and session summaries is publicly available in the repository referenced in the Data Availability Statement. Importantly, robots Xplorer A–C share the same author-developed baseline stack, so inter-robot variability reflects physical differences (mechanical tolerances, calibration drift, and sensor noise) rather than software inconsistency.

### 5.1. Route Planning Performance

Route planning via ComputeRoute was evaluated in 17 Test 1 sessions across the three robots. Planning time averaged 17.8 ms (range: 7.8–47.1 ms), with the variation attributable to graph complexity at the time of planning—sessions with semantic penalties active required slightly longer planning (30–47 ms) than those with a clean graph (8–16 ms). All planning times remained well below the 2 s timeout configured for the Route Server.

On a clean graph (no semantic penalties), the Route Server consistently selected the direct route (0 → 2 → 7) with a cost of 5.7 (geometric distance only). When semantic penalties were present, the same start–goal pair produced costs ranging from 42 to 107, and in several instances the Route Server selected alternative routes through nodes 3 or 5, demonstrating that penalty-weighted edge scoring actively influences route selection.

### 5.2. Navigation Performance

**Figure 8:**
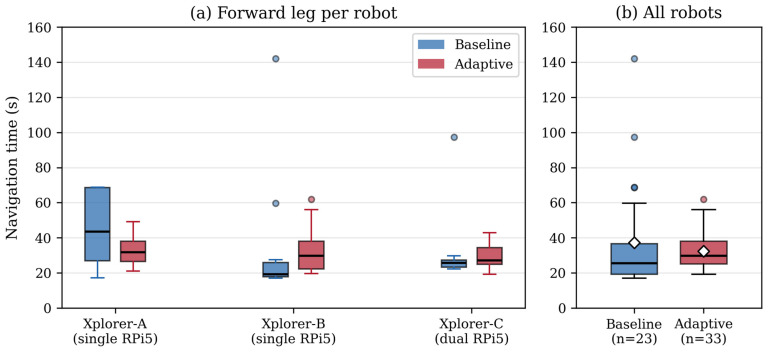
Forward leg navigation time distribution. (**a**) Per robot, baseline (blue) vs. adaptive (red). The adaptive condition shows substantially lower variability across all platforms. (**b**) Aggregated across all robots (n = 23 baseline, n = 33 adaptive). The diamond markers indicate mean values. The baseline outliers at 97.4 s and 142.0 s correspond to AMCL recovery stalls.

The aggregated forward navigation time was 37.3 ± 31.1 s for the baseline condition and 32.5 ± 11.0 s for the adaptive condition. The median values were closer (25.4 s baseline, 29.8 s adaptive), and a Mann–Whitney U test confirmed that the difference in central tendency was not statistically significant (U = 294, *p* = 0.157; see Section 5.2.1). Two baseline runs exhibited navigation times of 97.4 s and 142.0 s, caused by temporary AMCL recovery cycles that stalled the free-space planner; no adaptive run exceeded 62 s (Figure 8).

The more practically relevant observation is the reduction in navigation time variability: the standard deviation decreased from 31.1 s (baseline) to 11.0 s (adaptive), a variance ratio of 7.92. Levene’s test indicated a marginally significant difference in dispersion (W = 3.14, *p* = 0.082). This pattern was consistent across all three robots (Table 8 and Table 9), suggesting that graph-based route planning via the Route Server produces more predictable navigation behavior than the free-space planner, which is susceptible to costmap-induced detours and localization recovery stalls.

The average velocity was 0.248 ± 0.097 m/s for the baseline and 0.229 ± 0.053 m/s for the adaptive, consistent with the MPPI configuration (vx_max = 0.5 m/s, typical cruising 0.30–0.45 m/s). The lower variability in the adaptive velocity (σ = 0.053 vs. σ = 0.097) is consistent with the AdjustSpeedLimit operation moderating velocity based on semantic metadata.

#### 5.2.1. Statistical Analysis

For the navigation time distributions the Shapiro–Wilk test was used to test for normality. Both the baseline (W = 0.670, *p* < 0.001) and adaptive (W = 0.897, *p* = 0.005) forward-leg distributions were significantly non-normal, consistent with the right-skewed shape due to the AMCL recovery outliers in the baseline condition. Non-parametric tests were therefore used for all subsequent comparisons.

The Mann–Whitney U on forward-leg for navigation times gave U = 294, *p* = 0.157, n_1_ = 23, n_2_ = 33 meaning that no statistically significant difference in central tendency between the baseline and adaptive conditions. The corresponding effect size was small (Cohen’s d = 0.22). The result is expected because the adaptive condition is not designed to reduce mean navigation time, but rather to improve route predictability through penalty-weighted planning. The similar median values (25.4 s baseline, 29.8 s adaptive) indicated that the mean difference reported above is justified primarily by two baseline outliers (97.4 s and 142.0 s) caused by AMCL recovery stalls.

**Figure 9:**
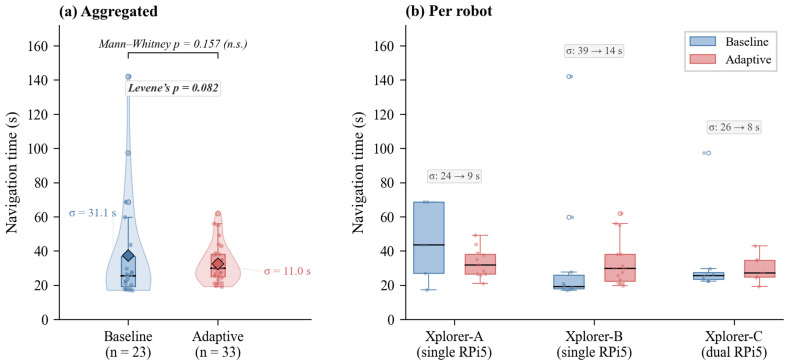
Statistical comparison of forward-leg navigation times (validated runs). (**a**) Aggregated distributions (baseline n = 23, adaptive n = 33) shown as violin plots with overlaid box plots and individual data points (diamonds indicate means). Both distributions were significantly non-normal (Shapiro–Wilk *p* < 0.005). The Mann–Whitney U test found no significant difference in central tendency (U = 294, *p* = 0.157). Levene’s test indicated a marginally significant difference in variance (W = 3.14, *p* = 0.082), with the baseline standard deviation nearly three times larger than the adaptive (31.1 s vs. 11.0 s). (**b**) Per-robot comparison showing consistent variance reduction across all three platforms, including Xplorer-C with its dual-RPi5 inference-offloading architecture.

Levene’s test gave W = 3.14 and *p* = 0.082. This means there was an almost significant difference in how much the navigation times varied between the two conditions. With adaptive routing the spread dropped a lot—the standard deviation went from 31.1 down to 11.0 (ratio ≈ 2.83, variance about 8 times smaller). Even though it did not quite reach the usual *p* < 0.05 cutoff, the same pattern appeared for all three robots and both leg directions (see Figure 9). The numbers in Table 8 and Table 9 confirm that graph-based route planning makes navigation times much more predictable.

Fisher’s exact test on the success rate (baseline 45/45 vs. adaptive 66/70) indicated *p* = 0.154, suggesting that the difference in success rates was not statistically significant at this sample size. All four adaptive failures were caused by AMCL drift on a single robot (Xplorer-B) and not because of the semantic pipeline or the Route Server.

#### 5.2.2. Success Rate

Baseline navigation achieved a 100% success rate (45/45 legs). Adaptive navigation achieved 94% (66/70 legs), with all four failures occurring on Xplorer-B. Failure analysis revealed that three failures were caused by AMCL drift during navigation (the robot deviated from the planned route and exceeded the 2.0 m XY error threshold), and one was caused by the path-gap issue (Section 3.6) prior to the implementation of the interpolation fix. No failures were attributable to the semantic pipeline or the Route Server logic.

#### 5.2.3. Prescan Overhead

The prescan phase (360° rotation + 35 s pipeline wait + heading realignment) required 11.3 ± 1.9 s of robot motion time, with the 35 s software wait dominating the total overhead. This time is reported separately and excluded from the navigation times in Table 8 and Table 9. The total wall-clock time for an adaptive forward leg (prescan + navigation) was therefore approximately 79 s (35 + 11.3 + 32.5), compared to 37.3 s for a baseline forward leg. The prescan overhead is a current limitation discussed in Section 6.

### 5.3. Route Selection and Replanning

**Figure 10:**
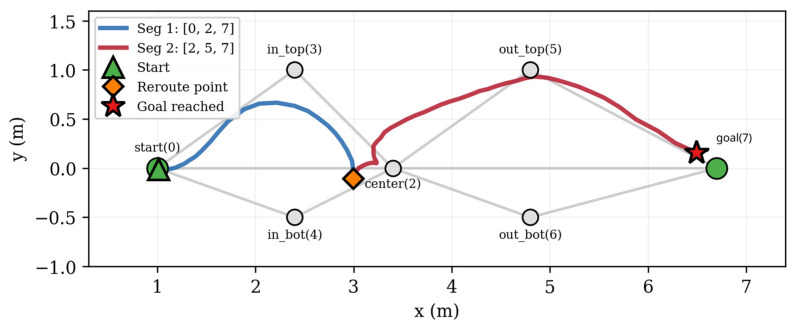
Trajectory of an adaptive forward leg (Xplorer-B) with a reroute event. The initial route (0, 2, 7) (blue) was replanned mid-navigation to (2, 5, 7) (red) after penalty changes were detected. The reroute point (orange diamond) marks where FollowPath was canceled and a new ComputeRoute was issued.

Seven distinct route configurations were observed at the forward leg Table 10. The direct route (0 → 2 → 7) was the most frequent (17/33 = 52%), selected when the total penalties on the center–goal edges were lower than the alternative paths through the upper nodes. Routes through node 3 (upper entry) and node 5 (upper exit) were selected when penalties on the direct 2 → 7 and 0 → 2 edges exceeded those on the alternative paths. No route through the lower nodes (4, 6) was observed, indicating that the lower path consistently had higher aggregate penalties in the experimental configuration (Figure 10).

The return legs showed a similar diversity with routes through nodes 5 and 3 appearing when penalties favored them, and Xplorer-C exhibited the highest route diversity including routes through both the upper (3, 5) and direct (2) nodes.

**Figure 11:**
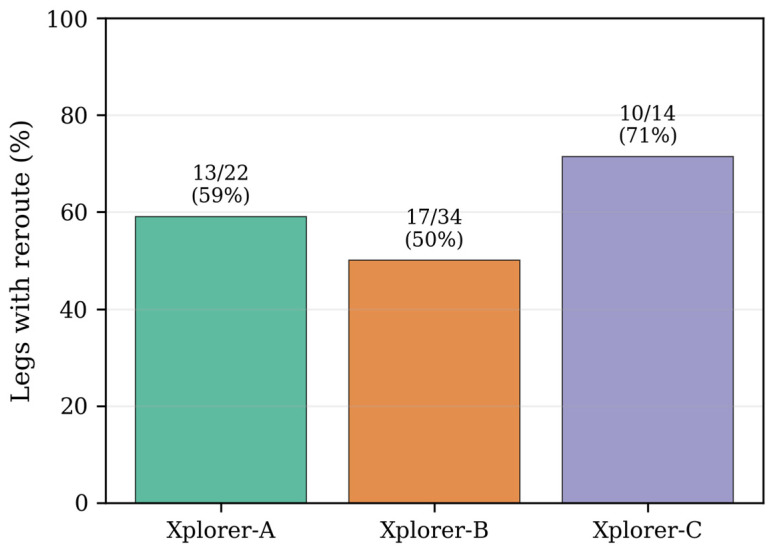
The percentage of adaptive navigation legs with at least one reroute event per robot. Xplorer-C achieved the highest reroute rate (71%), potentially benefiting from reduced CPU contention for more reliable penalty monitoring.

The adaptive condition produced 42 replanning events across 40 of 70 adaptive legs (57%). Replanning occurred on all three robots: Xplorer-A (59% of legs), Xplorer-B (50%), and Xplorer-C (71%) like in Figure 11.

The replanning mechanism was triggered when the edge penalty changes exceeded the threshold δ = 2.0 during navigation. A typical replanning sequence involved the robot starting on the direct route, the semantic pipeline updating penalties mid-navigation as new detections accumulated, and the Route Server selecting a shorter remaining route that bypassed the highly penalized edges. Many of the replanning events resulted in route truncation (e.g., (0, 2, 7) → (2, 7)) rather than route switching, reflecting the small graph size where partial routes dominate.

### 5.4. Semantic Awareness

#### 5.4.1. Objects Detected

The ASF pipeline detected 9 distinct object classes across all adaptive sessions, with potted plants being the most frequent (535 cumulative detections), followed by benches (140), dining tables (99), persons (85), and chairs (73). Bottles (43), cups (4), bowls (3), and beds (3) were also detected but with lower frequency.

The number of objects detected per prescan varied by robot and session: Xplorer-A detected 35.3 ± 17.7 objects per prescan (32.0 ± 16.6 valid), Xplorer-B detected 30.7 ± 16.9 (26.9 ± 14.0 valid), and Xplorer-C detected 10.7 ± 3.9 (10.0 ± 3.4 valid). The lower detection count on Xplorer-C is attributed to a different operational environment (different session, fewer objects physically present) rather than to the dual-RPi5 architecture, since the camera-LiDAR pipeline functions identically regardless of where the YOLO node executes.

People were detected in sessions on all three robots: Xplorer-A (4.1 ± 3.3 per prescan), Xplorer-B (2.1 ± 2.5), and Xplorer-C (0.6 ± 0.8). These were real individuals passing through the corridor during experiments, confirming that the pipeline handles dynamic objects in authentic, non-staged conditions.

#### 5.4.2. Minimum Object Distance

The minimum distance between the robot trajectory and the nearest detected semantic object was 0.4 ± 0.8 m for the baseline forward legs and 0.3 ± 0.3 m for the adaptive forward legs. This result, where the adaptive condition shows a slightly shorter minimum distance than the baseline, requires careful interpretation. The baseline condition uses the costmap-only free-space planner, which avoids physical obstacles but has no awareness of semantic objects—it may or may not pass near detected objects, depending on the costmap state and the planner decisions. The adaptive condition actively routes around high-penalty edges, but the graph topology constrains the available paths, and in a compact environment (a graph spanning approximately 5.7 m), all routes necessarily pass within the proximity radius (1.5 m) of at least some detected objects. The key benefit of semantic routing is at the route level (avoiding concentrations of high-penalty objects) rather than at the centimeter level.

### 5.5. System Resource Utilization

On the single-RPi5 platforms (Xplorer-A and B), the combined system load (Nav2 + YOLO + semantic pipeline + system monitoring) consumed approximately 85% of the CPU. The YOLO26n detector running via NCNN achieved 5.5 ± 0.7 Hz (approximately 167 ± 21 ms per inference), well within the 15 Hz rate limiter configured for the detector node. The inference rate was bottlenecked by the NCNN computation on the Cortex-A76 cores rather than by the rate limiter, confirming that the rate limiter serves as a ceiling for faster platforms rather than a constraint on the RPi5.

**Figure 12:**
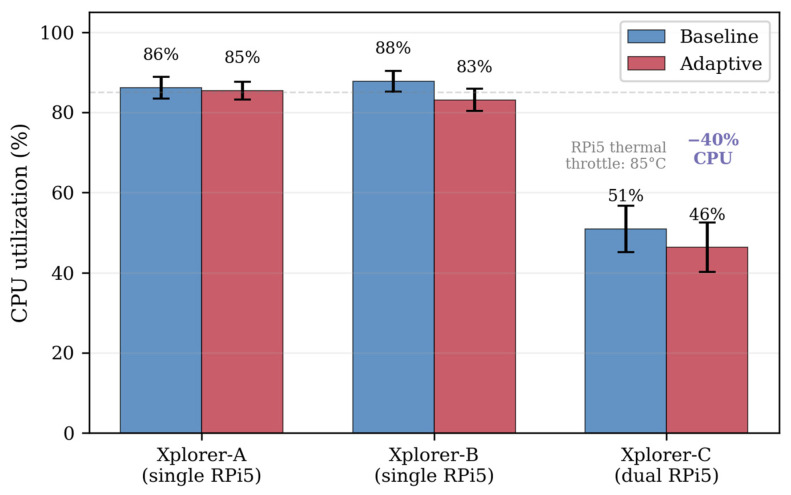
The CPU utilization across three compute architectures. Xplorer-A and B (single RPi5) operate at approximately 85% of the CPU. Xplorer-C (dual RPi5 with inference offloading) operates at approximately 48%, a reduction of 40 percentage points.

Xplorer-C, with the YOLO inference offloaded to a second RPi5 connected via Ethernet, operated at 47–51% of the CPU—a reduction of approximately 40 percentage points compared to the single-board platforms (Figure 12). This substantial reduction in CPU utilization leaves significant headroom for additional computational tasks (e.g., higher-resolution mapping, additional perception pipelines, or multi-robot coordination). The YOLO diagnostics topic was not captured on Xplorer-C’s navigation RPi5 because the topic ‘/yolo26/diagnostics’ messages originate on the remote compute unit; however, the semantic pipeline received detections normally over the ROS 2 DDS network, as confirmed by the 10.7 objects detected per prescan.

The CPU temperature remained below 70 °C on all platforms (the RPi5 thermal throttling threshold: 85 °C), indicating an adequate thermal margin for sustained operation. Xplorer-C exhibited slightly higher temperatures (69.3 °C vs. 65.8 °C), attributable to the different chassis ventilation rather than the computational load (Table 11).

### 5.6. Cross-Platform Consistency

All three robots achieved comparable navigation performance despite the hardware differences (Xplorer-C: different drivetrain geometry and dual-RPi5 architecture). The forward navigation times were 33.1 ± 8.6 s (A), 33.3 ± 13.9 s (B), and 29.7 ± 8.0 s (C) for the adaptive condition, with overlapping mean ± standard deviation ranges across all platforms. Xplorer-C achieved a 100% reroute rate (7/7 adaptive forward legs triggered at least one replanning event) and a 100% success rate (30/30 legs), suggesting that the reduced CPU contention from inference offloading may contribute to more reliable navigation behavior. Here, a reroute event denotes the FollowPath cancelation followed by a new ComputeRoute request triggered by a penalty delta exceeding δ.

The consistent performance across platforms validates the runtime self-configuration design of the ASF pipeline (Section 3.2): all sensor-specific parameters were acquired from ROS topics, and the URDF-derived TF transforms correctly resolved the different frame geometries of Xplorer-C without code modification.

## 6. Discussion

### 6.1. Key Findings

The experimental results demonstrate that the proposed semantic-aware route planning pipeline operates reliably on the edge hardware (RPi5, CPU-only) in a real indoor environment with three heterogeneous robot platforms. Five principal findings result from the data.

First, the Angular Sector Fusion pipeline provides lightweight object localization sufficient for route-level decisions. With a computational cost below 1 ms per detection and 2–5% of the CPU at 3 Hz, ASF adds a negligible and accepted overhead to the navigation stack. The pipeline was set to detect 9 object classes across all sessions, including dynamic objects (persons) in non-staged conditions, confirming its applicability to authentic indoor environments.

Second, the Nav2 Route Server successfully uses runtime semantic penalties to differentiate routes. Seven distinct route configurations were observed in the adaptive forward legs, with the Route Server selecting alternative paths through the upper nodes (3, 5) when penalties on the direct route exceeded those on the alternatives. The route planning completed in under 50 ms in all cases, responding well to the real-time requirements.

Third, the replanning mechanism was validated experimentally, with 42 reroute events observed across 57% of the adaptive legs and all three robots. This demonstrates that the penalty monitoring and route cancelation logic functions correctly on the real hardware, responding to the penalty changes that accumulate as the semantic pipeline processes new detections during navigation.

Fourth, even though the adaptive routing did not significantly reduce the average navigation time (Mann–Whitney *p* = 0.157), it greatly reduced the variability (standard deviation dropped from 31.1 s to 11.0 s for the forward legs; Levene’s test W = 3.14, *p* = 0.082). This consistent reduction in spread across all three robots is a really useful result: the graph-based routing produces much more predictable navigation times than free-space planning, which often gets hit by costmap detours and AMCL recovery stalls that cause occasional very long times. The fact that the average time did not change much actually fits the system’s design goal—to improve route quality and predictability rather than just raw speed.

Fifth, delegating the inference to a separate compute unit (Xplorer-C with dual-RPi5 setup) dropped the navigation CPU usage from 85% to 48%—a 40 percentage point reduction. This shows the system architecture makes modular deployment straightforward: perception and navigation can run on completely different hardware connected via ROS to DDS, with no code changes at all. The ASF pipeline automatically configures itself from the topics, no matter where they physically come from.

### 6.2. Experimental Design Considerations and Limitations

The design considerations were as follows: the study is intentionally designed as an ablation comparison between the baseline and adaptive navigation, executed as repeated trials across robots and sessions to reflect real-world indoor variability. This structure favors ecological validity (non-staged corridor conditions and naturally occurring obstacles) while maintaining procedural control through the automated execution, explicit validation criteria, and identical graph/controller settings across modes.

Measurement bias was mitigated as follows: the experimental design addresses potential measurement bias through several structural choices. First, the independent variable (navigation mode) and the dependent variables (navigation time, distance, success) are measured by different subsystems: the navigation mode is determined by the test protocol, while outcomes are recorded by the Nav2 action servers (success/failure), wheel odometry (distance, velocity), AMCL (XY error at goal), and wall-clock timers (duration)—each operating independently. Second, cross-platform replication across three robots with different drivetrains, calibrations, and mechanical tolerances provides a form of independent validation: consistent results across platforms (Table 8 and Table 9) would be unlikely if the outcomes were an artifact of a single measurement chain. Third, the automated test framework applies identical validation criteria (Section 4.3) uniformly across all conditions, eliminating observer bias. Fourth, the semantic pipeline’s object positions could not be independently verified by an external system (e.g., motion capture) in the current experimental setup; however, the route-level effect of semantic penalties is validated indirectly through the observed route diversity (7 distinct route configurations, Table 10) and replanning events (42 across 70 adaptive legs), which would not occur if the penalty annotations were inconsistent or incorrect. The absence of external ground truth for object localization accuracy is acknowledged as a limitation; future work will incorporate an overhead camera or motion capture system for independent position validation.

The limitations were as follows: the dataset is collected in a single-corridor environment with a fixed 7-node route graph; therefore, the generalization to larger or topologically different indoor spaces should be assessed in future work. Trials are repeated within sessions, and despite blocking by robot and day, the residual correlations may exist due to shared environment dynamics (e.g., traffic patterns). To mitigate this, we report dispersion metrics and focus the comparisons on the validated runs under consistent success criteria. Future work will extend the evaluation to additional buildings and include mixed-effects statistical modeling with the robot and session as random factors.

Nevertheless, the single-environment design was a deliberate choice to isolate the effect of semantic routing from the environmental confounders: by keeping the corridor layout, lighting conditions, and object types constant across 115 navigation legs, the comparison between the baseline and adaptive modes reflects the differences attributable to the semantic pipeline rather than to environmental variation. The use of three robots with two distinct compute architectures further strengthens internal validity. The generalization to larger topologies and different building types remains a priority for future work (Section 7).

### 6.3. Comparison with Related Work

The primary advance over Alqobali et al. is the integration of semantic information into a map production approach, rather than using it only for map annotation (Table 12). Their system detects objects uses and places them on a semantic map but does not use this information to influence navigation decisions. Our system closes this loop: the detected objects generate penalties on graph edges, the Route Server selects penalty-aware routes, and the replanning mechanism adapts routes during navigation when the semantic state changes.

A secondary advance is the transition from simulation to real hardware validation across multiple platforms. While the simulation provides controlled repeatability, it does not capture the localization drift, sensor noise, and CPU contention that characterize the real deployments. Our 115 navigation legs across three robots with a 97% success rate provide confidence that the system operates reliably under authentic conditions.

The measured YOLO throughput of 5.5 FPS on the RPi5 (CPU-only, NCNN backend) is comparable to the estimated 4 FPS of Alqobali et al. on the RPi4, despite using a more recent model (YOLO26n vs. YOLOv3). This confirms that the CPU-only inference on the ARM platforms remains in the 4–8 FPS range without GPU or NPU acceleration, and that the semantic pipeline must be designed to function at this detection rate—a constraint that our 3 Hz fusion rate and temporal tracking satisfy.

The dominant approach in the Nav2 ecosystem for incorporating semantic information into navigation is through custom costmap layers, where detected objects modify cell costs in the local or global costmap, influencing the free-space planner’s path at the cell level. Examples include custom obstacle layers that inject YOLO detections as costmap obstacles, and social navigation layers that create cost zones around detected persons. Our approach operates at a fundamentally different level: instead of modifying costmap cells, we annotate graph edges with semantic penalties and let the Route Server select among topologically distinct routes. This route-level integration has two advantages: (i) it preserves Nav2’s proven costmap-based local avoidance entirely unchanged, avoiding the risk of costmap corruption from false detections, and (ii) it enables route-level decisions (e.g., avoiding an entire corridor with people) that cell-level cost modifications cannot easily achieve, since the free-space planner may still select the same topological corridor even with elevated cell costs. The tradeoff is that route-level semantics require a predefined navigation graph, whereas costmap approaches work with any free-space planner. The two approaches are complementary rather than competing and combining them. Therefore graph-level route selection with cell-level semantic costmap refinement is a natural extension for future work.

### 6.4. Limitations

The following limitations should be considered when interpreting the results.

The 2D LiDAR plane constraint means that objects above or below the scan height (approximately 16 cm at Xplorer-A) produce no range returns; the ASF pipeline relies on the assumption that objects detected in the camera image intersect the LiDAR plane, which holds for common indoor objects (chairs, persons, potted plants) but may fail for wall-mounted or elevated objects.

The camera field of view (approximately 50°, covering 70 of 503 LiDAR rays) requires a 360° prescan rotation to observe the full environment. This prescan overhead (35 s pipeline wait + 11 s rotation) is the dominant time cost of the adaptive condition and makes the approach best suited to semi-static environments where the prescan investment is amortized over multiple navigation legs. Reducing the pipeline wait time through optimized save/reload cycles or incremental graph updates is a priority for future work.

In practice, the 35 s pipeline wait is a conservative setting that guarantees full graph annotation convergence on the RPi5; on faster hardware or with incremental graph updates, this delay could be substantially reduced. The prescan approach is well suited to environments where the semantic layout changes slowly relative to the navigation frequency—such as office corridors, warehouses, and hospital hallways where furniture and equipment remain in place for hours or days. In highly dynamic environments (e.g., crowded lobbies with continuous pedestrian flow), the prescan model would need to be replaced by continuous perception during navigation, which is identified as a priority in future work (Section 7).

The navigation graph used in the experiments contains 7 nodes and 10 bidirectional connections, spanning approximately 5.7 m. This compact topology limits route diversity: the direct route [0 → 2 → 7] was selected in 52% of the forward legs, and no route through the lower nodes (4,6) was observed. Larger graphs in more complex environments would provide a greater opportunity for the Route Server to differentiate routes and for semantic penalties to have a more pronounced effect on navigation behavior.

The minimum object distance metric showed no improvement in the adaptive condition compared to the baseline (0.3 m vs. 0.4 m). This is a consequence of the compact graph topology where all routes pass within the edge proximity radius (1.5 m) of at least some detected objects. The benefit of semantic routing operates at the route level (avoiding high-penalty concentrations) rather than at the centimeter level, and would be more apparent in larger environments with spatially separated route alternatives.

All four navigation failures occurred on a single robot (Xplorer-B) and were caused by AMCL drift, not by the semantic pipeline or Route Server. This highlights that localization reliability remains the primary practical constraint for autonomous indoor navigation on edge hardware, independent of the semantic layer.

The semantic routing pipeline depends on accurate localization at three levels: (i) the ASF pipeline uses the TF2 map→odom transform to project detected objects into the map frame—localization errors directly shift the estimated object positions, potentially causing penalties to be assigned to incorrect graph edges; (ii) the ComputeRoute request uses the explicit AMCL pose as the start position (use_start = True), so a drifted pose may result in the robot being associated with a wrong graph node; and (iii) the FollowPath controller tracks a dense path that assumes alignment between the planned and actual robot positions. In our experiments, AMCL operated reliably in the majority of trials (111/115 validated), and the four failures all involved temporary localization recovery stalls on Xplorer-B during which the map → odom transform diverged beyond the 2.0 m validation threshold. Importantly, once AMCL recovered, the semantic pipeline resumed correct operation without manual intervention. The reported navigation performance (97% success rate, route diversity, replanning behavior) should therefore be interpreted as representative of conditions where AMCL is functional, which is the expected operating regime for indoor environments with pre-built occupancy grid maps. Environments with poor localization conditions (feature-sparse corridors, highly reflective surfaces, or frequent kidnapping) would degrade both the baseline and semantic navigation equally, since both depend on the same AMCL + EKF state estimation stack.

The GeoJSON metadata constraint in the Nav2 Route Server 1.3.10 (only scalar float values accepted) required a dual-graph architecture: a full semantic graph for analysis and a flattened Nav2-compatible graph for the Route Server. This is a practical limitation of the current Nav2 release rather than a fundamental architectural constraint, and it may be resolved in future versions.

### 6.5. Practical Findings for the Nav2 Community

Several implementation details discovered during this work may benefit practitioners deploying the Nav2 Route Server on edge hardware:

The Route Server must be deployed as a standalone lifecycle node, outside the Nav2 lifecycle manager, to allow independent configuration, activation, and graph reloading without affecting the navigation stack. Dynamic graph replacement via the ‘set_route_graph’ service enables quasi-dynamic semantic routing without restarting the Route Server process.

The ComputeRoute action requires ‘use_start = True’ with an explicit AMCL pose as the start position after any AMCL reinitialization. Using ‘use_start = False’ (letting the Route Server determine position from TF) can produce stale results when the map → odom transform has not yet converged, generating paths that start far from the robot’s actual position.

The path-gap phenomenon—where the Route Server generates a dense path starting from the nearest graph node rather than from the robot’s position—can be mitigated by prepending interpolated waypoints from the robot to the path start when a gap exceeding a configurable threshold is detected. This correction is essential for reliable FollowPath execution, particularly for return legs where the robot may be associated with a non-adjacent graph node.

The C++ NCNN lifecycle node pattern (model loading at configure, inference at activate, rate limiter, subscriber-gated diagnostics) provides a reproducible deployment template for YOLO inference on ARM edge platforms with a measured throughput of 5.5 Hz on the RPi5 Cortex-A76.

## 7. Conclusions

This paper presented a lightweight semantic-aware route planning system for indoor mobile robots, integrating monocular camera object detection with 2D LiDAR distance estimation on edge hardware through the Angular Sector Fusion (ASF) pipeline, and connecting the resulting semantic map to the Nav2 Route Server for penalty-weighted route selection.

The ASF pipeline achieves runtime self-configuration from standard ROS 2 topics and the URDF transform tree, requiring no hardcoded sensor parameters and enabling deployment across heterogeneous platforms without code modification. On the RPi5 (CPU-only), the pipeline adds less than 5% CPU overhead at a 3 Hz fusion rate, with the YOLO26n detector (C++ NCNN) operating at 5.5 ± 0.7 FPS (167 ± 21 ms per inference).

Experimental validation on 115 navigation legs across three robots (two single-RPi5 and one dual-RPi5 architecture) demonstrated a 97% overall success rate (100% baseline, 94% adaptive), with all adaptive failures attributable to AMCL drift rather than the semantic pipeline, with 42 replanning events observed across 57% of the adaptive legs. Even though adaptive routing showed no significant reduction in average time (Mann–Whitney *p* = 0.157), it greatly lowered the variability (standard deviation dropped from 31.1 s to 11.0 s; Levene’s test W = 3.14, *p* = 0.082). This produced much more predictable navigation behavior across all three robots. Inference offloading to a dedicated compute unit reduced navigation CPU utilization from 85% to 48%, confirming the system’s suitability for modular edge deployments.

The three principal contributions are: (C1) the ASF pipeline as a zero-parameter, auto-configuring fusion method requiring no learned components; (C2) an experimental validation of the Nav2 Route Server with runtime semantic penalties on physical robots, including replanning; and (C3) a practical characterization of deployment constraints including the GeoJSON metadata limitation, the path-gap phenomenon, and the ‘use_start’ requirement, documented for the Nav2 community.

### Future Work

Four directions are prioritized for future development. First, evaluation in more diverse and challenging indoor environments is planned: controlled dynamic scenarios with persons entering and leaving specific routes will validate the replanning mechanism under conditions designed to trigger full route switching rather than route truncation; additional environments including multi-room office layouts, open-plan workspaces with movable furniture, and a warehouse-like setting with narrow aisles will test the system’s adaptability to different spatial structures, object densities, and pedestrian traffic patterns. Second, deployment on larger navigation graphs is planned to evaluate scalability: a 20–30 node graph spanning multiple connected corridors within the FIIR building (~40 m path length), and a full-floor graph with 50+ nodes covering approximately 200 m of traversable path. These configurations will provide sufficient topological diversity for semantic penalties to produce measurably different route choices and will test the Route Server planning time under increased graph complexity. Third, integration with 3D depth cameras (e.g., RealSense™ D435i) will address the 2D LiDAR plane limitation and enable localization of elevated or wall-mounted objects. Fourth, the prescan overhead can be reduced through continuous incremental graph updates during navigation, eliminating the dedicated scan-and-wait phase.

## Figures and Tables

**Table 1:** Xplorer platform specifications.

Component	Xplorer-A/B	Xplorer-C
**Compute (navigation)**	Raspberry Pi 5, 16 GB, ARM Cortex-A76, Ubuntu 24.04	Same
**Compute (inference)**	Same RPi5 (shared)	2nd Raspberry Pi 5, 16 GB, Ethernet
**Camera**	Logitech C920 HD 1080 p, configured at 640 × 480, USB	Same
**2D LiDAR**	STL-19P (LD19), 360°, ∼503 rays, 10 Hz, range 0.02–25 m	Same
**IMU**	BNO055, 9-DoF, I2C interface	-
**Drivetrain**	4WD differential, goBILDA, 4× quadrature encoders (223 rpm)	4WD differential, 4× hall encoders (178 rpm)
**Motor Controllers**	2× RoboClaw 2 × 15A, UART packet serial	2× L298N + ESP32
**Power**	12 V Li-ion with BMS, RPi5 via PD 5 V	Same
**ROS distribution**	ROS 2 Jazzy	Same
**Navigation stack**	Nav2 1.3.10 (AMCL, MPPI controller, SmacPlannerHybrid)	Same
**Localization**	AMCL + EKF (robot localization), predict at 20 Hz	Same

**Table 2:** Modules introduced in this work and their ROS 2 interfaces and persisted artifacts.

Module (This Work)	Purpose	ROS 2 Inputs (Topics/Actions)	ROS 2 Outputs (Topics/Actions/Services)	Persisted Artifacts (Files)
**yolo26_cpp** **(C++ detector, lifecycle)**	Real-time object detection on RPi5 (local or offloaded)	Camera image stream (sensor_msgs/Image) *(topic name depends on bringup)*	/yolo26/detections (vision_msgs/Detection2DArray), /yolo26/diagnostics (std_msgs/String JSON)	(optional) model/config on disk (weights, ncnn params)
**semantic_localizer (ASF)**	Projects 2D detections to LiDAR angular sectors and localizes objects in map frame	/yolo26/detections, LaserScan *(topic auto-configured)*, TF2 transforms, /amcl_pose for map-frame traceability	Object updates to semantic map manager (internal), optional debug/log messages	—
**SemanticMapManager** **(persistent semantic map)**	Maintains object store with TTL/EMA and serializes stable objects	ASF localized objects (internal stream)	Semantic snapshot for tests/analysis	semantic_objects.geojson
**Graph annotator + dual-graph exporter**	Annotates edges with penalties/speed limits and exports Nav2-safe graph	Semantic map snapshot, base graph	Updated Route Server graph (Nav2-compatible)	Nav2-compatible graph GeoJSON + optional semantic graph (diagnostics)
**Route Server integration** **(Nav2)**	Semantic-aware route selection	ComputeRoute planning requests; penalty deltas	FollowPath execution; optional reroute triggers	Graph + route config snapshots in session dir
**Experimental suite v5.10 (test_semantic_navigation_v5_10.py)**	Automated Test 1–3 execution, validation, and logging	ComputeRoute (Test 1), baseline NavigateToPose, odom (cfg.odom_topic), /amcl_pose, /yolo26/diagnostics, BMS topic (battery), TF2	Per-run JSON logs; aggregated session summary; printed comparisons	route_config.yaml, per-run JSON, session_config.json, summary.json

**Table 3:** Sensor parameters relevant to the Angular Sector Fusion. All values are acquired at runtime from the ROS topics (CameraInfo, LaserScan) and the TF2 transform tree. The values shown correspond to the Logitech C920 at 640 × 480 and the STL-19P LiDAR as deployed on the Xplorer platforms.

Parameter	Camera (Logitech C920)	LiDAR (STL-19P)	Source
**ROS frame**	camera_link_optical	base_laser	URDF/TF2
**Position vs. base_link (x, y, z)**	(0.228, 0.0, 0.095) m	(0.222, 0.0, 0.160) m	TF2 static transform
**Focal length (f_x)**	687.54 px	—	CameraInfo.K0
**Principal point (c_x)**	308.11 px	—	CameraInfo.K2
**Resolution**	640 × 480 px	—	CameraInfo.width/height
**Horizontal FOV**	49.8° (±24.9°)	360°	Derived from f_x
**Angular resolution**	—	0.716° (~503 rays)	LaserScan.angle_increment
**Range limits**	—	0.02–25.0 m	LaserScan.range_min/max
**Update rate**	30 Hz (native)	10 Hz	Topic rate
**Effective processing rate**	15 Hz (rate-limited)	cached (latest scan)	Software config
**Rays covering camera FOV**	—	~70	Derived at runtime

**Table 4:** Object mobility classification with persistence parameters.

Category	Representative Classes	Base Penalty	Speed Limit	TTL (s)	Position Update
**Dynamic**	person, cat, dog	50.0	30%	60	Overwrite
**Static**	chair, bench, couch, tv, refrigerator	15.0	60%	∞ (permanent)	Fused (α = 0.7)
**Minor**	bottle, cup, book, remote	5.0	90%	120	Fused (α = 0.7)

**Table 5:** Navigation graph topology. All coordinates are in the map frame (meters).

Node	ID	Position (x, y)	Role	Connections
start	0	(1.0, 0.0)	Start terminal	→ 2, 3, 4
center	2	(3.4, 0.0)	Central hub	→ 0, 3, 4, 5, 6, 7
in_top	3	(2.4, 1.0)	Upper route entry	→ 0, 2
in_bot	4	(2.4, −0.5)	Lower route entry	→ 0, 2
out_top	5	(4.8, 1.0)	Upper route exit	→ 2, 7
out_bot	6	(4.8, −0.5)	Lower route exit	→ 2, 7
goal	7	(6.7, 0.0)	Goal terminal	→ 2, 5, 6

**Table 6:** Experimental test protocol.

Phase	Name	Description	Robot Movement
Test 0	Setup	Backup existing semantic map, reset via service call, wait for pipeline repopulation (60 s), regenerate Nav2-compatible graph, reinitialize AMCL	Convergence spin only
Test 1	ComputeRoute	Single route planning request (start → goal); validates Route Server functionality and route selection	None
Test 2	Baseline	NavigateToPose using SmacPlannerHybrid + MPPI; N runs, each with forward (start → goal) and return (goal → start) legs	Full navigation
Test 3	Adaptive	Semantic-aware navigation using Route Server; N runs with prescan, route computation, FollowPath with replanning	Full navigation + prescan

**Table 7:** Metrics collected per navigation leg.

Category	Metric	Unit	Collection Method
Navigation	Duration (excl. prescan)	s	Wall clock timer
	Distance traveled	m	Odometry integration
	XY error at goal	m	AMCL pose vs. goal coordinates
	Average velocity	m/s	Odometry
	Maximum velocity	m/s	Odometry
	Trajectory (map frame)	[x, y, t]	AMCL pose at 2 Hz
Routing	Route type	Up/Down/ Mix/DIRECT	identify_route()
	Planning time	ms	Wall clock timer
	Route cost	—	ComputeRoute result
	Reroute count	—	Replanning log
	Penalties per edge	—	Nav2 graph on disk
Semantic	Min distance to objects	m	Trajectory vs. semantic map
	Detected objects (count, classes)	—	semantic_objects.geojson
Perception	YOLO FPS	Hz	/yolo26/diagnostics @ 5 s
	YOLO inference time	ms	/yolo26/diagnostics @ 5 s
	Detections per frame	—	/yolo26/diagnostics @ 5 s
System	CPU utilization (total + per-core)	%	psutil @ 1 Hz
	Memory utilization	%	psutil @ 1 Hz
	CPU temperature	°C	sysfs thermal zone @ 1 Hz
	Battery delta	%	BMS topic

**Table 8:** Navigation performance summary for forward legs (validated runs only). Navigation time excludes the prescan overhead. Xplorer-C uses a dual-RPi5 architecture with YOLO inference offloaded to a dedicated compute unit (Section 5.5).

Robot	Condition	n	Nav Time (s)	Distance (m)	XY Error (m)	Reroutes	CPU (%)
Xplorer-A	Baseline	5	45.0 ± 23.5	6.7 ± 0.5	0.12 ± 0.06	0	85.3 ± 3.0
Xplorer-A	Adaptive	11	33.1 ± 8.6	6.9 ± 0.8	0.12 ± 0.06	0.7 ± 0.5	85.4 ± 2.3
Xplorer-B	Baseline	10	35.9 ± 39.5	7.3 ± 2.6	0.25 ± 0.09	0	86.7 ± 2.9
Xplorer-B	Adaptive	15	33.3 ± 13.9	7.0 ± 0.7	0.20 ± 0.08	0.5 ± 0.6	83.1 ± 3.1
Xplorer-C	Baseline	8	34.2 ± 25.7	6.4 ± 0.4	0.15 ± 0.07	0	50.9 ± 5.6
Xplorer-C	Adaptive	7	29.7 ± 8.0	6.2 ± 0.7	0.38 ± 0.51	1.0 ± 0.0	47.3 ± 6.8
All	Baseline	23	37.3 ± 31.1	6.8 ± 1.7	0.18 ± 0.09	0	73.9 ± 17.6
All	Adaptive	33	32.5 ± 11.0	6.8 ± 0.8	0.21 ± 0.25	0.7 ± 0.5	76.3 ± 15.8

**Table 9:** Navigation performance summary for return legs (validated runs only).

Robot	Condition	n	Nav Time (s)	Distance (m)	XY Error (m)	Reroutes
Xplorer-A	Baseline	5	35.0 ± 17.3	6.8 ± 0.5	0.04 ± 0.04	0
Xplorer-A	Adaptive	11	37.7 ± 12.6	7.1 ± 0.7	0.17 ± 0.07	0.5 ± 0.7
Xplorer-B	Baseline	9	39.9 ± 24.5	6.9 ± 0.6	0.08 ± 0.04	0
Xplorer-B	Adaptive	15	29.9 ± 12.1	6.9 ± 1.7	0.18 ± 0.09	0.6 ± 0.5
Xplorer-C	Baseline	8	35.8 ± 6.7	6.8 ± 0.7	0.07 ± 0.08	0
Xplorer-C	Adaptive	7	33.3 ± 13.0	6.1 ± 0.7	0.18 ± 0.06	0.3 ± 0.5
All	Baseline	22	36.9 ± 17.5	6.8 ± 0.6	0.07 ± 0.06	0
All	Adaptive	33	33.2 ± 12.4	6.8 ± 1.3	0.17 ± 0.08	0.5 ± 0.6

**Table 10:** Route diversity observed in adaptive forward legs (validated).

Route (Node Sequence)	Occurrences	Classification
[0, 2, 7]	17	Direct
[2, 7] (from reroute mid-navigation)	6	Direct (partial)
[0, 2, 5, 7]	3	Upper
[2, 5, 7] (from reroute)	3	Upper (partial)
[3, 2, 7]	2	Bottom(entry)
[0, 3, 2, 7]	1	Upper
[3, 2, 5, 7]	1	Upper

**Table 11:** System resource utilization per robot platform.

Metric	Xplorer-A	Xplorer-B	Xplorer-C
Architecture	Single RPi5	Single RPi5	Dual RPi5 (split)
YOLO node location	Local	Local	Remote (2nd RPi5)
CPU total—baseline	86.2 ± 2.8%	87.7 ± 2.6%	50.9 ± 5.8%
CPU total—adaptive	85.5 ± 2.2%	83.1 ± 2.8%	46.3 ± 6.2%
CPU temperature max	65.6 ± 0.9 °C	66.0 ± 0.6 °C	69.3 ± 4.2 °C
YOLO FPS	5.4 ± 0.5 Hz	5.6 ± 0.8 Hz	N/A (remote)
YOLO inference time	169.2 ± 16.8 ms	165.5 ± 23.8 ms	N/A (remote)

**Table 12:** Comparison with the closest related system.

Aspect	Alqobali et al. [2] (Sensors, 2024)	Yuan et al. [13] (Sensors, 2025)	Galindo et al. [15] (RAS, 2008)	This Work
**Environment**	Simulation (Gazebo)	Lab/industrial, static targets	Simulation + limited real	Real indoor (3 robots)
**Hardware**	RPi4 (4 GB) OAK-DPro—Luxonis	Industrial camera 720P + LSLIDAR TOF	Not specified (desktop)	RPi5 (16 GB) Logitech C920 HD
**Detector**	YOLOv3 (Python, OpenCV DNN)	Semantic segmentation	N/A	YOLO26n (C++ NCNN, lifecycle)
**Detector throughput**	~4 FPS (estimated)	Not reported	N/A	5.5 ± 0.7 FPS (measured)
**LiDAR**	RPLiDAR A1	LSLIDAR TOF (360°, 0.48°)	N/A	STL-19P (360°, 10 Hz)
**Fusion method**	Bounding box center + LiDAR	Contour inverse projection	N/A	ASF: angular sector + percentile
**Route planning**	None (costmap only)	None	Hierarchical task planner	Nav2 Route Server (PenaltyScorer)
**Semantic influence**	Map annotation only	Point cloud labeling only	Task planning from semantic map	Map + routing penalties + replanning
**Replanning**	Not implemented	N/A	N/A	Implemented (δ > 2.0 threshold)
**Reroute events observed**	N/A	N/A	N/A	42 events across 70 adaptive legs
**Cross-platform validation**	Single simulated robot	Single lab setup	Single robot	3 physical robots, 2 architectures
**Inference monitoring**	None	None	N/A	Integrated (/yolo26/diagnostics)
**Test automation**	Manual	Manual	Manual	Fully automated (v5.10, JSON output)
**Sensor auto-configuration**	Not reported	Manual calibration (homography matrix)	N/A	All parameters from ROS topics + URDF

## Data Availability

The data and software supporting the findings of this study are publicly available in the GitHub repository nav2-semantic-route-server at https://github.com/bogdan-abaza/nav2-semantic-route-server (accessed on 31 March 2026). These materials include the complete experimental dataset comprising 115 navigation legs across 27 sessions, per-run JSON logs, session summary files, navigation graph files, the source code for all developed modules, and the automated test framework (accessed on 31 March 2026).
